# Lactylation stabilizes TFEB to elevate autophagy and lysosomal activity

**DOI:** 10.1083/jcb.202308099

**Published:** 2024-08-28

**Authors:** Yewei Huang, Gan Luo, Kesong Peng, Yue Song, Yusha Wang, Hongtao Zhang, Jin Li, Xiangmin Qiu, Maomao Pu, Xinchang Liu, Chao Peng, Dante Neculai, Qiming Sun, Tianhua Zhou, Pintong Huang, Wei Liu

**Affiliations:** 1 Center for Metabolism Research, The Fourth Affiliated Hospital of Zhejiang University School of Medicine, and International School of Medicine, International Institutes of Medicine, Zhejiang University, Yiwu, China; 2 Department of Ultrasound Medicine, The Second Affiliated Hospital of Zhejiang University School of Medicine, Hangzhou, China; 3 National Center for Protein Science Shanghai, Institute of Biochemistry and Cell Biology, Shanghai Institutes of Biological Sciences, Chinese Academy of Sciences, Shanghai, China

## Abstract

The transcription factor TFEB is a major regulator of lysosomal biogenesis and autophagy. There is growing evidence that posttranslational modifications play a crucial role in regulating TFEB activity. Here, we show that lactate molecules can covalently modify TFEB, leading to its lactylation and stabilization. Mechanically, lactylation at K91 prevents TFEB from interacting with E3 ubiquitin ligase WWP2, thereby inhibiting TFEB ubiquitination and proteasome degradation, resulting in increased TFEB activity and autophagy flux. Using a specific antibody against lactylated K91, enhanced TFEB lactylation was observed in clinical human pancreatic cancer samples. Our results suggest that lactylation is a novel mode of TFEB regulation and that lactylation of TFEB may be associated with high levels of autophagy in rapidly proliferating cells, such as cancer cells.

## Teaser

Posttranslational modifications play a crucial role in regulating autophagy and lysosome–related transcription factor TFEB. Huang et al. find the increase in intracellular lactate induces TFEB lactylation. Lactylation at K91 protects TFEB from WWP2-mediated ubiquitination and proteasomal degradation, thereby enhancing lysosomal activity and autophagy flux.

## Introduction

Autophagy is a lysosome-dependent cellular catabolism that mediates the delivery of cellular components to lysosomes for degradation and recycling. Among the three known types of autophagy, macroautophagy (hereafter referred to as autophagy) is characterized by the formation of double-membraned autophagosomes mediated by a series of autophagy-related proteins. Increasing evidence indicates that autophagy dysregulation is closely related to multiple major human diseases, including neurodegenerations and cancer. However, regarding the role of autophagy in cancer, although the inhibitory effect and mechanism of autophagy on tumor development have been revealed, cancer cells typically exhibit active autophagy while rapidly growing and proliferating (Karantza-Wadsworth et al., 2007; Komatsu et al., 2007, 2010; Mathew et al., 2009; Tian et al., 2015). Even with sufficient nutrition, tumor cells present autonomously elevated basal autophagy (White, 2015; Yang et al., 2011), and inhibiting autophagy can inhibit tumor growth (Rao et al., 2014; Santanam et al., 2016; Yang et al., 2018). These findings suggest that cancer cells utilize autophagy to survive microenvironmental pressure and promote malignancy (Degenhardt et al., 2006; Levy et al., 2017), supporting the concept that autophagy defects lead to cell malignant transformation, whereas in established cancer, autophagy endows cancer cells with invasiveness and chemotherapy resistance (Galluzzi et al., 2015). Nevertheless, hitherto, little is known about the mechanisms by which tumor cells acquire and maintain high autophagy.

Transcription factor EB (TFEB) is a central regulatory factor for the expression of autophagy and lysosomal genes. Loss of TFEB disrupts autophagosome formation and lysosome biogenesis (Sardiello et al., 2009; Settembre et al., 2011), while TFEB overexpression promotes the degradation of numerous autophagic cargoes (Nezich et al., 2015; Settembre et al., 2011, 2013). Current studies indicate that TFEB activation is mainly regulated by posttranslational modifications, including phosphorylation and acetylation (Pena-Llopis et al., 2011; Settembre et al., 2011; Wang et al., 2020). mTORC1-mediated phosphorylation plays a core role by retaining TFEB in the cytoplasm, and mTORC1 inactivation drives the dephosphorylation and nuclear translocation of TFEB to initiate target gene transcription (Puertollano et al., 2018). Recent studies also show that the transcription of TFEB itself may involve a positive feedback regulation (Settembre et al., 2013), and E3 ubiquitin ligase STIP1 homology and U-Box containing protein 1 (STUB1) targets TFEB for ubiquitin-proteasomal degradation (Sha et al., 2017). Interestingly, in human cancers including pancreatic cancer and lung cancer, despite the aberrant hyperactivation of mTORC1, TFEB expression and activity are upregulated (Bertozzi et al., 2021; Giatromanolaki et al., 2015; Perera et al., 2015).

One of the most prominent features of rapidly proliferating cells is the dysregulation of cellular metabolism. Even under aerobic conditions, these cells reprogram glucose metabolism by limiting ATP synthesis primarily to glycolysis, producing high levels of lactate (Hanahan and Weinberg, 2011; Vander Heiden et al., 2009). Lactate concentration is ∼1.0 mM in human muscle and arterial blood, but can reach up to 40 mM in cancers (Cheetham et al., 1986; Walenta et al., 2000). Recent studies indicate that cancer cells may exploit lactate to fulfill a diverse array of functions. Under hypoxia, lactic acid produced by glycolysis binds to the oxygen regulatory protein NDRG3 and suppresses its degradation, so the increased NDRG3 can boost cell growth (Lee et al., 2015). Lactic acid also directly binds mitochondrial antiviral-signaling (MAVS) protein and inhibits pattern recognition receptor-mediated innate immunity (Zhang et al., 2019b). In addition, cancer cells use lactic acid as a carbon source for the TCA cycle to maintain cell metabolism (Faubert et al., 2017). Tumor-derived lactate curbs the infiltration and activity of natural killer cells and T cells (Brand et al., 2016), and triggers the M2-like polarization of tumor-associated macrophages (Colegio et al., 2014). More importantly, it has recently been found that lactate-driven histone lactylation serves as an epigenetic modification that directly stimulates gene transcription from chromatin (Zhang et al., 2019a). High-resolution mass spectrometry (MS) analysis shows that lysine lactylation may target a variety of proteins involved in diverse cellular processes (Wan et al., 2022).

Here, we identified the lactylation of TFEB while exploring the molecular mechanism of elevated autophagy in cancer cells. We found that lactate-induced lactylation of TFEB at the lysine 91 (K91) site protects TFEB from E3 ubiquitin ligase WWP2-mediated ubiquitination and proteasomal degradation, leading to increased lysosomal activity and autophagy flux. In human primary pancreatic ductal adenocarcinoma (PDA) samples, we observed enhanced lactylation of TFEB.

## Results

### Lactate promotes autophagy and lysosome biogenesis

**Figure 1.**
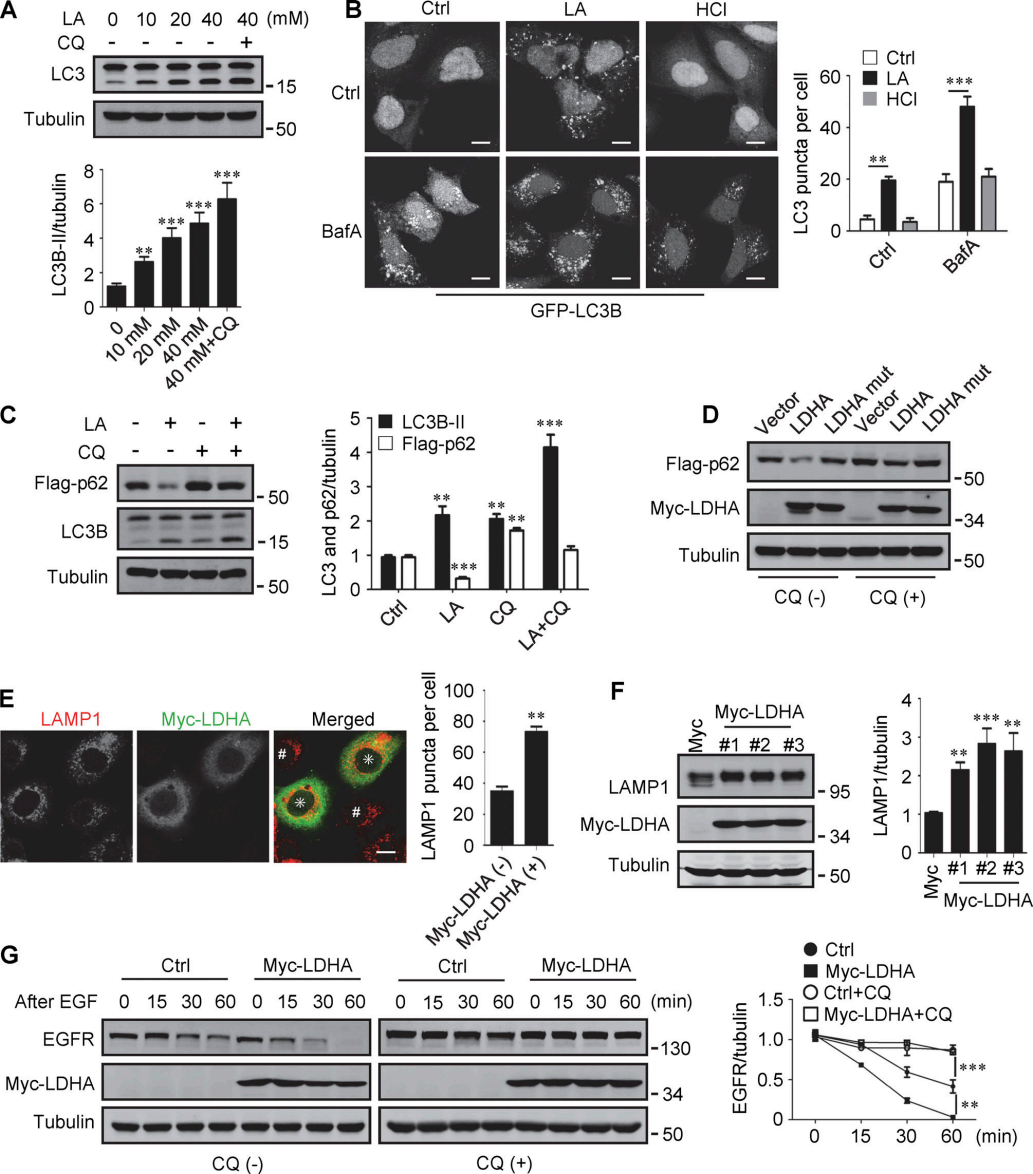
**Lactate promotes autophagy and lysosome biogenesis. (A)** Western blot of LC3 in HeLa cells treated with different concentrations of lactic acid (LA) with or without CQ. The quantitative data from three independent experiments represent mean ± SEM. **(B)** Distribution of GFP-LC3 in HEK293 cells stably expressing GFP-LC3. Cells were treated with lactic acid or 0.3 μM HCl, which caused the same pH change in the medium, with or without V-ATPase inhibitor bafilomycin A1 (BafA). Scale bars, 10 μm. The quantitative data are mean ± SEM (*n* = 30 cells). **(C)** Flag-p62 and LC3 levels in HEK293 cells stably expressing Flag-p62. Cells were treated with or without lactic acid and CQ. The quantification data from three independent experiments are presented as mean ± SEM. **(D)** Flag-p62 levels in HEK293 cells stably expressing Flag-p62. Cells were transfected with Myc-tagged WT-LDHA or inactive LDHA mutant with or without CQ treatment. **(E)** Immunostaining of LAMP1 in HeLa cells with or without Myc-LDHA expression. Cells that express Myc-LDHA are indicated with * and cells that do not express Myc-LDHA are indicated with #. Scale bars, 10 μm. The quantitative data are presented as mean ± SEM (*n* = 30 cells). **(F)** LAMP1 expression in three HeLa cell lines stably expressing Myc-LDHA. The quantification data from three independent experiments are mean ± SEM. **(G)** Degradation of EGFR in WT HeLa or HeLa cells stably expressing Myc-LDHA in the presence or absence of CQ. The quantitative data of three independent experiments are presented as mean ± SEM. **P < 0.01, ***P < 0.001. All molecular weights are in kD. Source data are available for this figure: SourceData F1.

**Figure S1.**
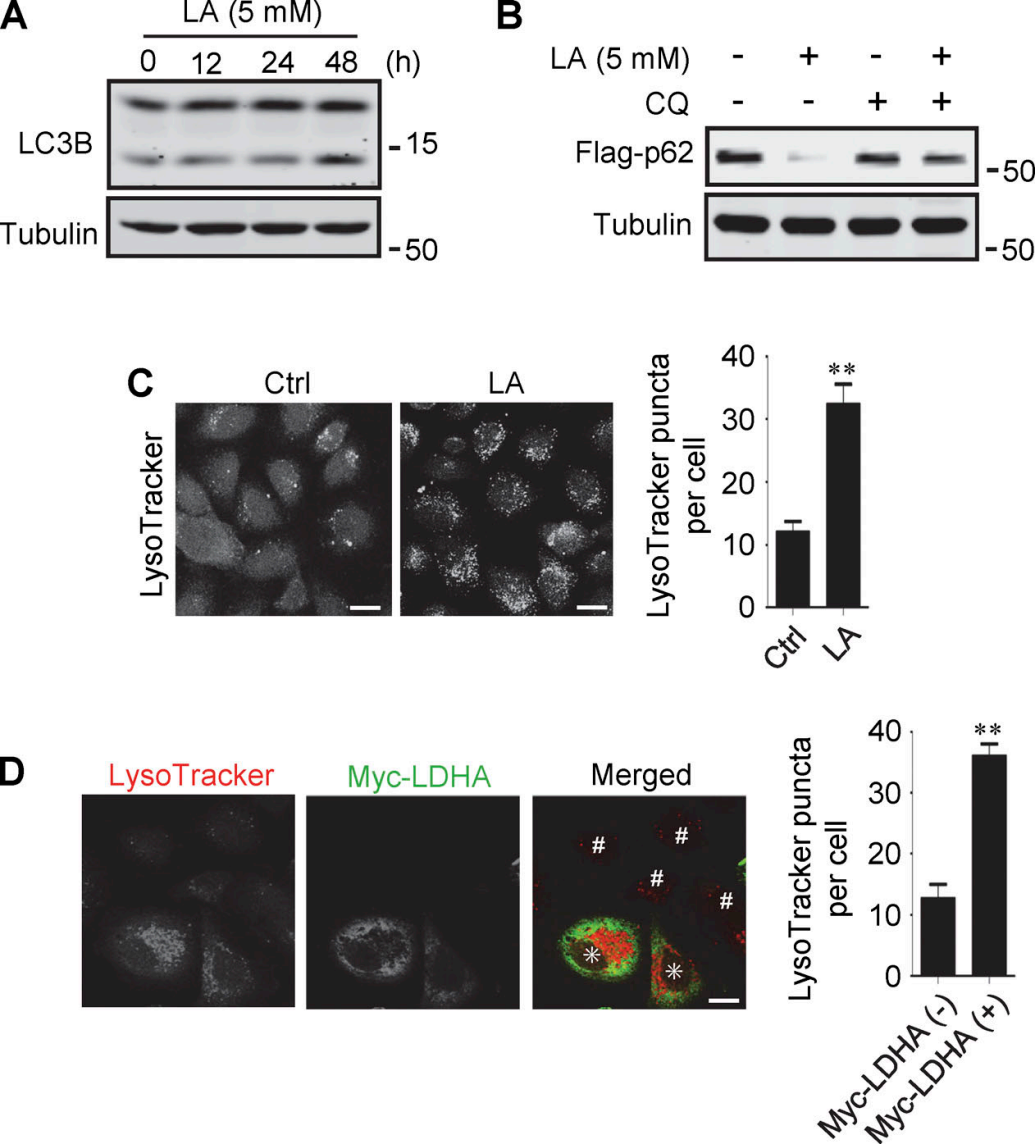
**Lactate promotes autophagy and lysosome biogenesis. (A)** Western blot analysis of LC3B expression in PANC cells treated with 5 mM lactic acid (LA) for the indicated time duration. **(B)** Flag-p62 levels in transiently transfected PANC cells treated with 5 mM lactic acid with or without CQ for 24 h. **(C)** Representative LysoTracker staining of HeLa cells after lactic acid treatment. The quantitative data are from 60 cells of three independent experiments and presented as mean ± SEM; **P < 0.01. **(D)** Representative LysoTracker staining of HeLa cells transfected with LDHA. The quantitative data are from 30 cells of three independent experiments and presented as mean ± SEM; **P < 0.01. Cells with LDHA expression are indicated with * while cells that do not express LDHA are indicated with #. **P < 0.01. All molecular weights are in kD. Scale bars: 20 μm in C, 10 μm in D. Source data are available for this figure: SourceData FS1.

Lactate has been reported to activate or inhibit autophagy in different cells (Fedotova et al., 2022; Xia et al., 2017). We found that treatment of HeLa cells with 10–40 mM lactic acid for 24 h or PDA cell line pancreatic cancer (PANC) cells with 5 mM lactic acid for 48 h increased intracellular LC3-II (Fig. 1 A and Fig. S1 A). In cells stably expressing GFP-LC3B, lactic acid, but not hydrochloric acid (HCl) that gave rise to equivalent pH change, significantly promoted the formation of GFP-LC3 puncta (Fig. 1 B). In addition, lactic acid treatment reduced the levels of cellular p62 protein, which was prevented by adding a lysosome inhibitor chloroquine (CQ) (Fig. 1 C and Fig. S1 B). The reduction in p62 was also observed in cells overexpressing wild-type (WT) but not enzymatically inactive lactate dehydrogenase A (LDHA) (Zhao et al., 2013) (Fig. 1 D). These results suggest that lactate fosters autophagy flux by stimulating the formation of autophagosomes. Interestingly, we noticed that LDHA overexpression led to an increase in LAMP1 puncta (Fig. 1 E), along with an increase in LAMP1 protein levels (Fig. 1 F). Because both lactic acid treatment and LDHA overexpression raised significantly the number of LysoTracker-labeled structures (Fig. S1, C and D), we analyzed the processing of epidermal growth factor receptor (EGFR) to determine lysosomal degradation activity. Expectedly, overexpression of LDHA expedited EGFR degradation in cells after EGF treatment (Fig. 1 G). Taken together, these results suggest that lactate can activate autophagy and lysosome biogenesis.

### Lactate upregulates TFEB levels

**Figure 2.**
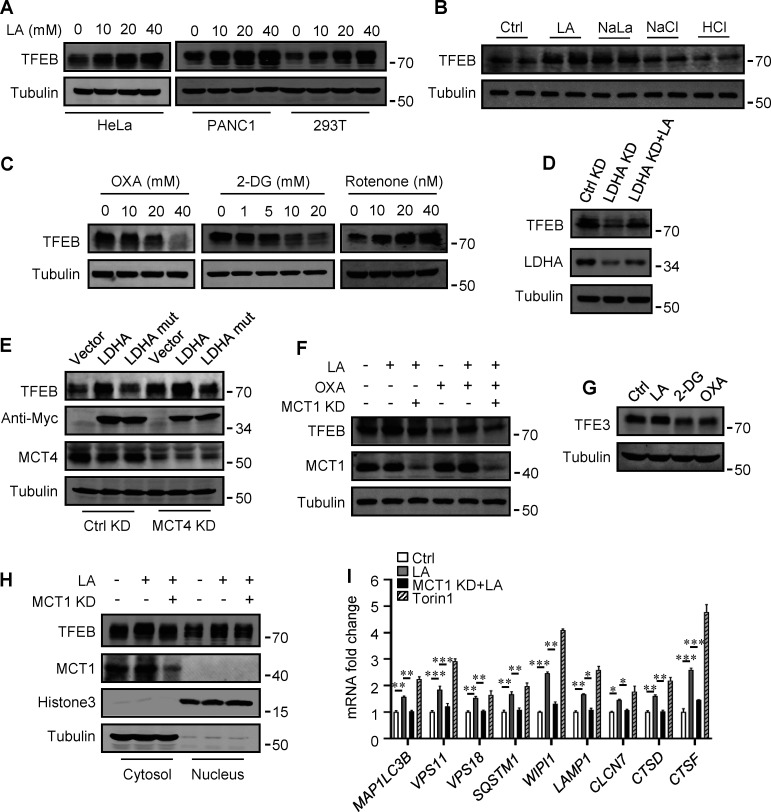
**Lactate upregulates TFEB protein levels. (A)** TFEB levels in HeLa, PANC1, and 293T cells after lactic acid (LA) treatment for 24 h. **(B)** TFEB expression in HeLa cells treated with 20 mM lactic acid, 20 mM sodium lactate (NaLa), 20 mM NaCl, or 0.3 μM HCl for 24 h. **(C)** TFEB levels in HeLa cells after 2-DG, OXA, or rotenone treatment for 24 h. **(D)** TFEB and LDHA expression in HeLa cells cultured with control or LDHA siRNA for 72 h and lactic acid for 24 h. **(E)** TFEB expression in HeLa cells cultured with control or MCT4 siRNA for 48 h followed by transfection with Myc-LDHA or inactive Myc-LDHA mutant for 24 h. **(F)** TFEB levels in HeLa cells cultured with control or MCT1 siRNA for 48 h followed OXA or lactic acid treatment for 24 h. **(G)** TFE3 expression in Hela cells after 24 h lactic acid, 2-DG, or OXA treatment. **(H)** TFEB and MCT1 levels in the cytoplasm and nucleus of fractionated HeLa cells. Cells were cultured with control or MCT1 siRNA for 48 h and lactic acid for 24 h. Tubulin and histone3 were used as cytoplasmic and nuclear markers, respectively. **(I)** RT-qPCR analysis of the expression of TFEB target genes in Hela cells. Cells were cultured with control or MCT1 siRNA for 48 h, then lactic acid or Torin 1 for 24 h. The data from three independent experiments are presented as mean ± SEM; *P < 0.05, **P < 0.01, ***P < 0.001. All molecular weights are in kD. Source data are available for this figure: SourceData F2.

**Figure S2.**
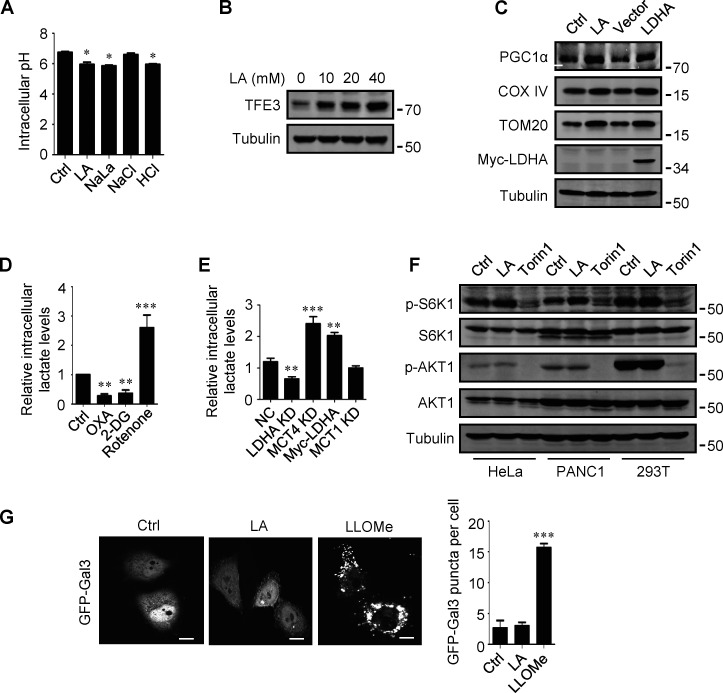
**Lactate upregulates TFEB. (A)** Intracellular pH levels of HeLa cells treated with the indicated chemicals. The data are presented as mean ± SEM (*n* = 3); *P < 0.05. **(B)** TFE3 expression in HeLa cells treated with different concentrations of lactic acid (LA). **(C)** The expression of PGC1α in HeLa cells treated with lactic acid or transfected with LDHA. **(D and E)** Intracellular lactate levels in HeLa cells treated with OXA, 2-DG, or rotenone (D), or cultured with LDHA, MCT4, or MCT1 siRNA, or transfected with Myc-LDHA (E). The data are presented as mean ± SEM (*n* = 3); **P < 0.01, ***P < 0.001. NC, negative control. **(F)** Western blot analysis of S6K1 and AKT1 phosphorylation in HeLa, PANC1, and 293T cells treated with lactic acid or Torin 1. **(G)** Representative images showing the distribution of GFP-Galectin three in transfected HeLa cells after 24 h lactic acid or 30 min LLOMe treatment. The quantitative data are from 30 cells of three independent experiments and presented as mean ± SEM; ***P < 0.001. Scale bars: 10 μm. All molecular weights are in kD. Source data are available for this figure: SourceData FS2.

The promoting effect of lactate on autophagosome formation and lysosome biogenesis indicates its potential impact on TFEB activity. Interestingly, we found that lactic acid treatment dose-dependently increased TFEB protein levels in HeLa cells, HEK293T cells, and PDA cell line PANC cells (Fig. 2 A). In addition, sodium lactate, instead of NaCl or HCl causing the same pH change (Fig. S2 A), also increased TFEB protein (Fig. 2 B). These results suggest that lactate may specifically affect the expression of TFEB protein. To corroborate this, we treated cells with different chemicals including the LDHA inhibitor sodium oxamate (OXA), the non-metabolizable glucose analog 2-deoxy-D-glucose (2-DG), and rotenone, which uncouples complex 1 from the electron transport chain and forces ATP generation via glycolysis. We found that treatments that led to reduced lactate production reduced TFEB, while treatments that promoted lactate production increased TFEB levels (Fig. 2 C). Consistently, knocking down LDHA reduced TFEB (Fig. 2 D) and overexpressing LDHA, but not the inactive LDHA mutant, increased TFEB (Fig. 2 E). Furthermore, knockdown (KD) of monocarboxylate transporter 4 (MCT4), a principal contributor to lactate efflux in highly glycolytic cells, raised TFEB protein levels and augmented TFEB accumulation led by LDHA overexpression (Fig. 2 E). It is worth noting that the addition of lactic acid reversed the decrease in TFEB caused by LDHA KD or OXA treatment, and the effect of lactate was offset by the KD of MCT1 responsible for lactate uptake (Fig. 2, D and F). These observations highlight the role of intracellular lactate. Interestingly, the cellular level of TFE3 protein, another member of the microphthalmia/transcription factor E family, also showed an increase in lactate-treated cells, while it decreased in cells treated with 2-DG and OXA that reduced lactate production (Fig. 2 G and Fig. S2 B). Finally, through subcellular fractionation of cells treated with lactic acid, we detected an increase in nuclear TFEB (Fig. 2 H), as well as enhanced expression of autophagy and lysosome-related TFEB target genes (Fig. 2 I), confirming that the increased TFEB expression is associated with an increase in cellular TFEB activity. The increased expression of transcription coactivators PGC1α and mitochondrial proteins COX IV and TOM20 in cells treated with lactic acid and in cells transfected with LDHA further supports this (Fig. S2 C), as PGC1α gene is a unique target of TFEB and plays a crucial role in mitochondrial biogenesis (Austin and St-Pierre, 2012). Using a lactate colorimetric/fluorometric assay kit, we determined that KD/overexpression of relevant genes and the chemicals used to manipulate lactate synthesis or transport did indeed correspondingly alter intracellular lactate levels (Fig. S2, D and E).

The mTORC1 and protein kinase B (AKT) signaling pathways are key negative regulators of TFEB. To clarify whether the increase in TFEB activity induced by lactate is due to mTORC1 or AKT inactivation, we examined the phosphorylation of mTORC1 substrate S6K1 and AKT1. We found that lactic acid treatment slightly increased S6K1 phosphorylation and did not significantly affect the phosphorylation of AKT1 (Fig. S2 F). Furthermore, we observed the cellular distribution of GFP-labeled Galectin-3 (Gal3), which is a recognized marker of damaged endomembranes (Paz et al., 2010). When cells treated with the lysosomotropic compound LLOMe exhibited a punctate distribution of GFP-Gal3, cells treated with lactic acid showed a diffusion distribution of GFP-Gal3 in the cytoplasm and nucleus, similar to untreated cells (Fig. S2 G).

### Lactate inhibits WWP2-mediated ubiquitination and proteasomal degradation of TFEB

**Figure S3.**
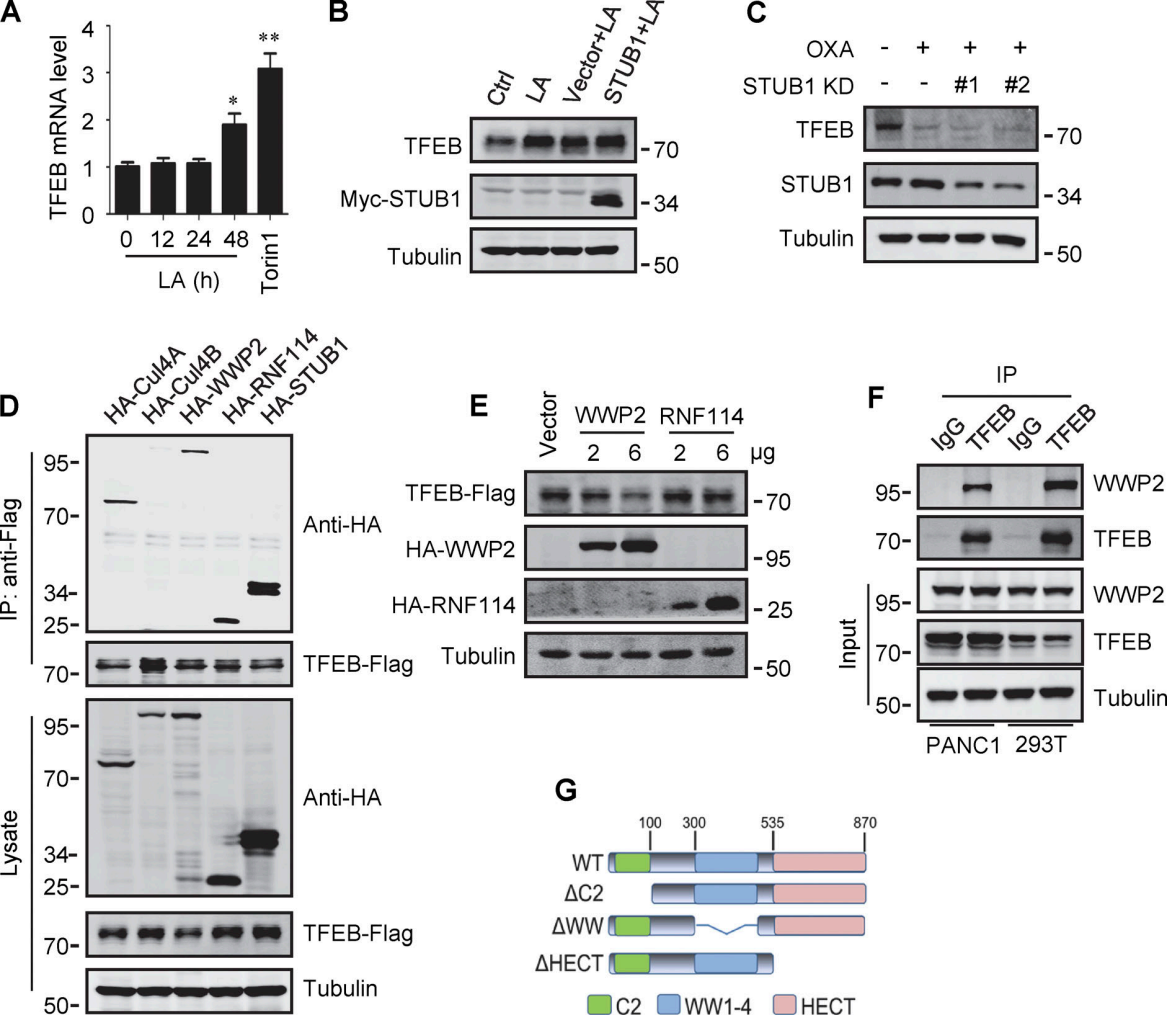
**Lactate inhibits TFEB ubiquitination and proteasomal degradation. (A)** Expression of TFEB mRNA in HeLa cells treated with lactate acid (LA) or Torin 1. Data are presented as mean ± SEM (*n* = 3 independent experiments); *P < 0.05, **P < 0.01. **(B)** TFEB levels in Hela cells transfected with STUB in the presence or absence of lactate acid. **(C)** TFEB expression in Hela cells transfected with STUB siRNA in the presence or absence of OXA. **(D)** Coprecipitation of potential E3 ligases with TFEB-Flag. HEK293 cells stably expressing TFEB-Flag were transfected with HA-tagged Cullin4A, Cullin4B, WWP2, RNF114, or STUB1. TFEB-Flag immunoprecipitated (IP) with anti-Flag was blotted with anti-HA. **(E)** TFEB-Flag levels in HEK293 cells co-transfected with HA-WWP2 or HA-RNF114. **(F)** Coprecipitation of endogenous WWP2 with TFEB. TFEB was immunoprecipitated from PANC1 cells and HEK293T cells using anti-TFEB, and the precipitates were blotted with anti-WWP2. IgG was used as a control for TFEB antibody. **(G)** Schematic diagram of WWP2’s domains and its distinct domain deletion mutants. All molecular weights are in kD. Source data are available for this figure: SourceData FS3.

**Figure 3.**
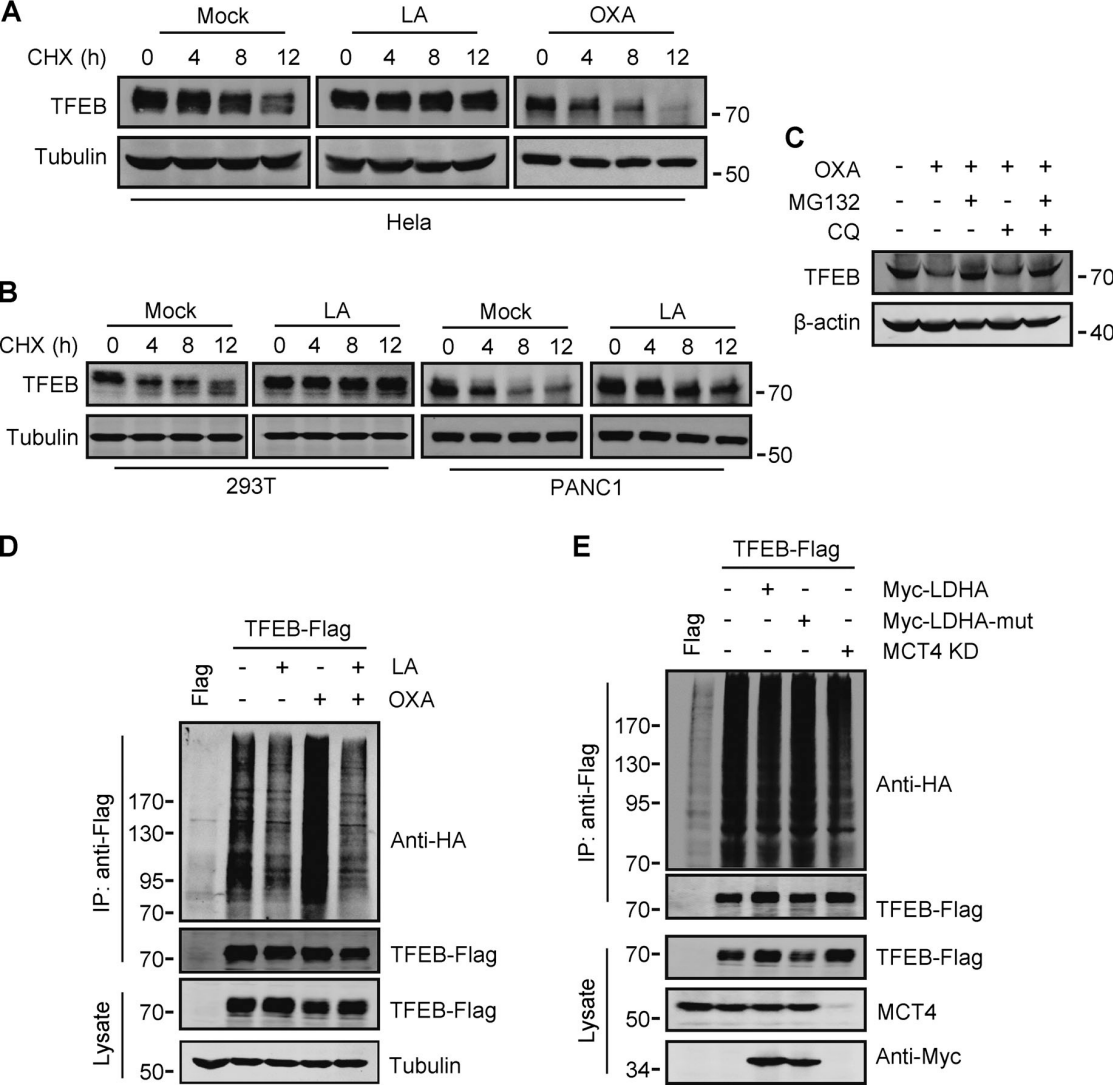
**Lactate inhibits TFEB ubiquitination and proteasomal degradation. (A and B)** Western blot analysis of TFEB in HeLa cells (A) and PANC1 and 293T cells (B) treated with lactic acid (LA) or OXA for the indicated duration in the presence of CHX. **(C)** TFEB levels in HeLa cells treated with OXA for 24 h in the presence or absence of CQ or MG132 for 6 h. **(D and E)** Ubiquitination of TFEB-Flag in HeLa cells expressing HA-ubiquitin. Cells were transfected with HA-ubiquitin and TFEB-Flag or empty Flag-vector. After 24 h, the cells were treated with lactic acid or OXA. **(D)** Cells expressing HA-ubiquitin were cultured for 48 h with or without MCT4 siRNA, and then transfected with TFEB-Flag and Myc-LDHA or inactive Myc-LDHA mutant for 24 h (E). TFEB-Flag was immunoprecipitated (IP) using anti-Flag and blotted with anti-HA. All molecular weights are in kD. Source data are available for this figure: SourceData F3.

To investigate the potential mechanism by which lactate upregulates TFEB, we measured TFEB mRNA expression. Surprisingly, TFEB mRNA levels only increased in cells exposed to high concentrations of lactic acid for extended periods of time (Fig. S3 A). This result suggests that lactic acid may have a stabilizing effect on TFEB protein. Indeed, in the presence of the protein synthesis inhibitor cycloheximide (CHX), the degradation of TFEB protein in HeLa was blunted by lactic acid treatment and accelerated by OXA treatment (Fig. 3 A). Consistent results were obtained in PANC1 cells and HEK293T cells (Fig. 3 B). The proteasome inhibitor MG132, instead of CQ, effectively prevented the degradation of TFEB stimulated by OXA (Fig. 3 C), supporting that intracellular TFEB is mainly degraded through the proteasome pathway (Sha et al., 2017). In line with this, TFEB ubiquitination was inhibited in lactate-treated cells and enhanced in OXA-treated cells (Fig. 3 D). Moreover, knocking down MCT4 or overexpressing LDHA instead of its inactivated mutant also reduced TFEB ubiquitination (Fig. 3 E). Based on these results, we propose that lactate inhibits TFEB degradation through the ubiquitin–proteasome system, thereby increasing cellular TFEB levels.

Previously, it was shown that the chaperone-dependent E3 ubiquitin ligase STUB1 targets TFEB for degradation (Sha et al., 2017). Surprisingly, overexpression of STUB1 did not reduce the accumulation of lactate-induced TFEB in HeLa cells (Fig. S3 B). In addition, the depletion of STUB1 did not offset the strong degradation of TFEB by OXA treatment (Fig. S3 C). Seeking novel E3 ubiquitin ligases that may be involved in lactate-induced TFEB stabilization, we employed immunoprecipitation combined with MS to screen for TFEB interacting proteins in HEK293T cells stably expressing TFEB-Flag. Among the proteins coprecipitated with TFEB-Flag (Table S1), several established E3 ubiquitin ligases were identified, including STUB1. Coprecipitation experiments affirmed the interaction of TFEB-Flag with exogenous STUB1, Cullin4A, WWP2, and RNF114, but not Cullin4B (Fig. S3 D). It is worth noting that relatively low HA-WWP2 expression markedly reduced TFEB-Flag in cell lysates, while higher STUB1 expression did not produce this effect (Fig. S3 D). Then, by comparing cells transfected with WWP2 and RNF114, we confirmed that overexpression of WWP2 but not RNF114 dose-dependently reduced TFEB levels (Fig. S3 E).

**Figure 4.**
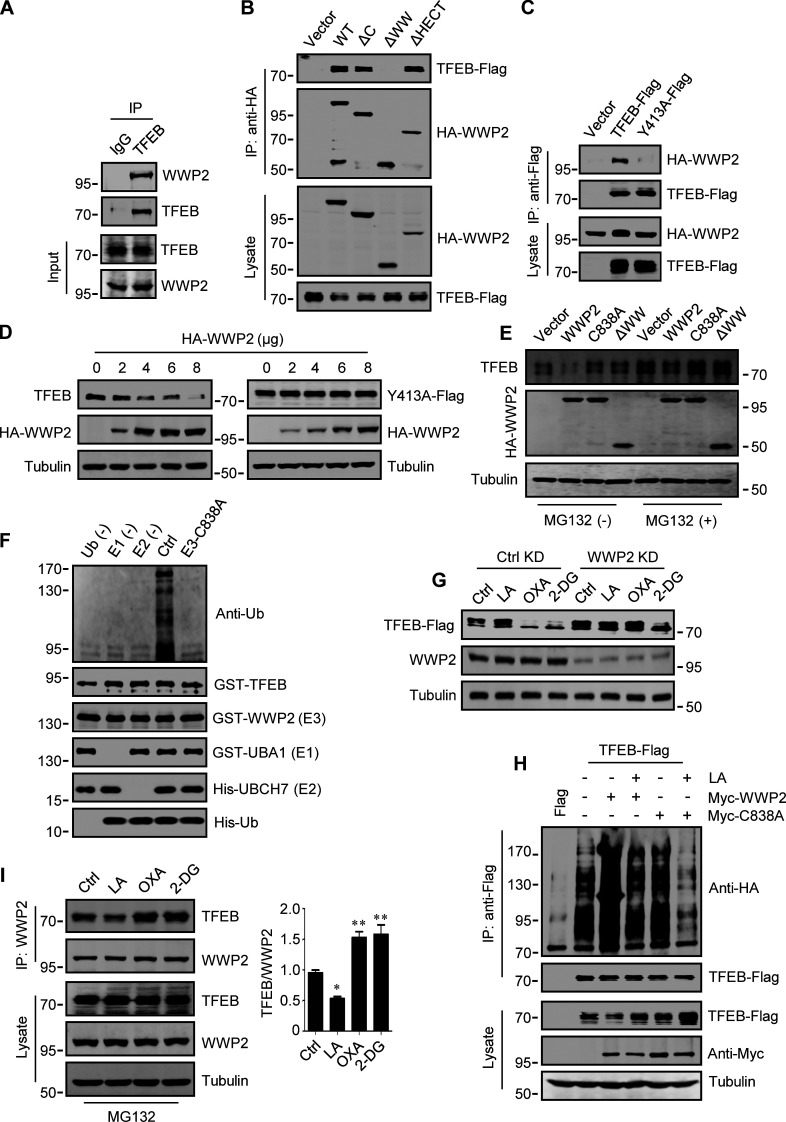
**WWP2 mediates lactate-regulated TFEB ubiquitination and proteasomal degradation. (A)** Coprecipitation of WWP2 with TFEB. TFEB immunoprecipitated (IP) from HeLa cells was blotted with anti-WWP2. IgG was used as a control for anti-TFEB. **(B)** Coprecipitation of TFEB-Flag with HA-WWP2. TFEB-Flag and HA-tagged WWP2 were cotransfected into HEK293 cells. After 24 h, HA-WWP2 was immunoprecipitated using anti-HA and analyzed by western blotting using anti-Flag. **(C)** Coprecipitation of HA-WWP2 with TFEB-Flag. HEK293 cells were transfected with HA-WWP2 and TFEB-Flag. After 24 h, TFEB-Flag was immunoprecipitated with anti-Flag and analyzed using anti-HA. **(D)** Effect of WWP2 expression on TFEB and TFEB-Y413A protein levels. Different amounts of HA-WWP2 plasmids were transfected into HeLa cells with or without TFEB-Y413A-Flag. After 24 h, the expression of endogenous TFEB in cells without TFEB-Y413A-Flag transfection and the transfected TFEB-Y413A-Flag were analyzed using anti-TFEB and anti-Flag, respectively. **(E)** TFEB levels in cells expressing WWP2 mutants. HeLa cells were transfected with HA-tagged WWP2. After 24 h, cells were treated with or without MG132 for 6 h and TFEB expression was analyzed. **(F)** In vitro ubiquitination assay of purified GST-TFEB. GST-TFEB was incubated with purified His-tagged ubiquitin (Ub) and UBA1 (E1) and GST-tagged UBCH7 (E2) and WWP2. The reactions were analyzed by western blotting using anti-ubiquitin. **(G)** Effect of WWP2 KD on TFEB expression. HEK293 cells with stable TFEB-Flag expression were cultured with control or WWP2 siRNA for 48 h, then treated as indicated, and TFEB-Flag expression were analyzed using anti-Flag. **(H)** Ubiquitination of TFEB-Flag in cells. HEK293 cells expressing HA-ubiquitin were transfected with TFEB-Flag and Myc-tagged WWP2. After 24 h, cells were treated with or without lactic acid (LA). Immunoprecipitated TFEB-Flag was blotted with anti-HA. **(I)** Effect of lactic acid on TFEB–WWP2 interaction. HeLa cells were treated as indicated in the presence of MG132. Immunoprecipitated WWP2 was blotted with anti-TFEB. The quantitative data of three independent experiments are presented as mean ± SEM. *P < 0.05, **P < 0.01. All molecular weights are in kD. Source data are available for this figure: SourceData F4.

WWP2 belongs to the E3 ligases family whose members contain a WW domain that binds to the substrate PPxY motif (Martin-Serrano et al., 2005). First, we proved that endogenous TFEB can coprecipitate endogenous TFEB (Fig. 4 A and Fig. S3 F). Then, using the constructed truncated WWP2 mutants (Fig. S3 G), we found that the WW domain of WWP2, but not its C2 or homologous to E6AP C-terminus domain, is necessary for TFEB interaction (Fig. 4 B). Interestingly, we found a PPGY sequence at the C-terminus of TFEB, where changing Y413 in PPGY to alanine led to a complete loss of interaction between TFEB and WWP2 (Fig. 4 C). Next, we investigated the effect of TFEB–WWP2 interaction on cellular TFEB protein levels. Overexpression of WWP2 reduced TFEB in a dose-dependent manner, while TFEB-Y143A expression was not affected at all (Fig. 4 D). The reduction of TFEB was averted in MG132-treated cells (Fig. 4 E), supporting the proteasome degradation of TFEB. In addition, overexpression of WWP2-∆WW lacking the ability to bind TFEB or WWP2-C838A lacking ubiquitin ligase activity (Maddika et al., 2011) also did not reduce TFEB levels (Fig. 4 E). Furthermore, in vitro ubiquitination assays showed that purified recombinant TFEB was strongly ubiquitinated in the presence of purified ubiquitin, E1, E2, and WWP2 but not WWP2-C838A (Fig. 4 F). These results therefore suggest that WWP2 is an E3 ubiquitin ligase of TFEB and mediates the proteasomal degradation of TFEB.

Finally, we determined the role of WWP2 in lactate-induced TFEB stabilization. In cells stably expressing TFEB-Flag, silencing WWP2 prevented TFEB-Flag degradation caused by OXA or 2-DG treatment (Fig. 4 G). Overexpression of WWP2 but not WWP2-C838A strongly promoted TFEB-Flag ubiquitination and the addition of lactate completely eliminated this effect (Fig. 4 H). In addition, the interaction between TFEB and WWP2 in cells was weakened under lactate treatment and enhanced under OXA or 2-DG treatment (Fig. 4 I). Taken together, these results suggest that lactate inhibits WWP2–TFEB interactions, thereby preventing WWP2-mediated TFEB ubiquitination and proteasomal degradation.

### Lactylation of TFEB at K91

**Figure S4.**
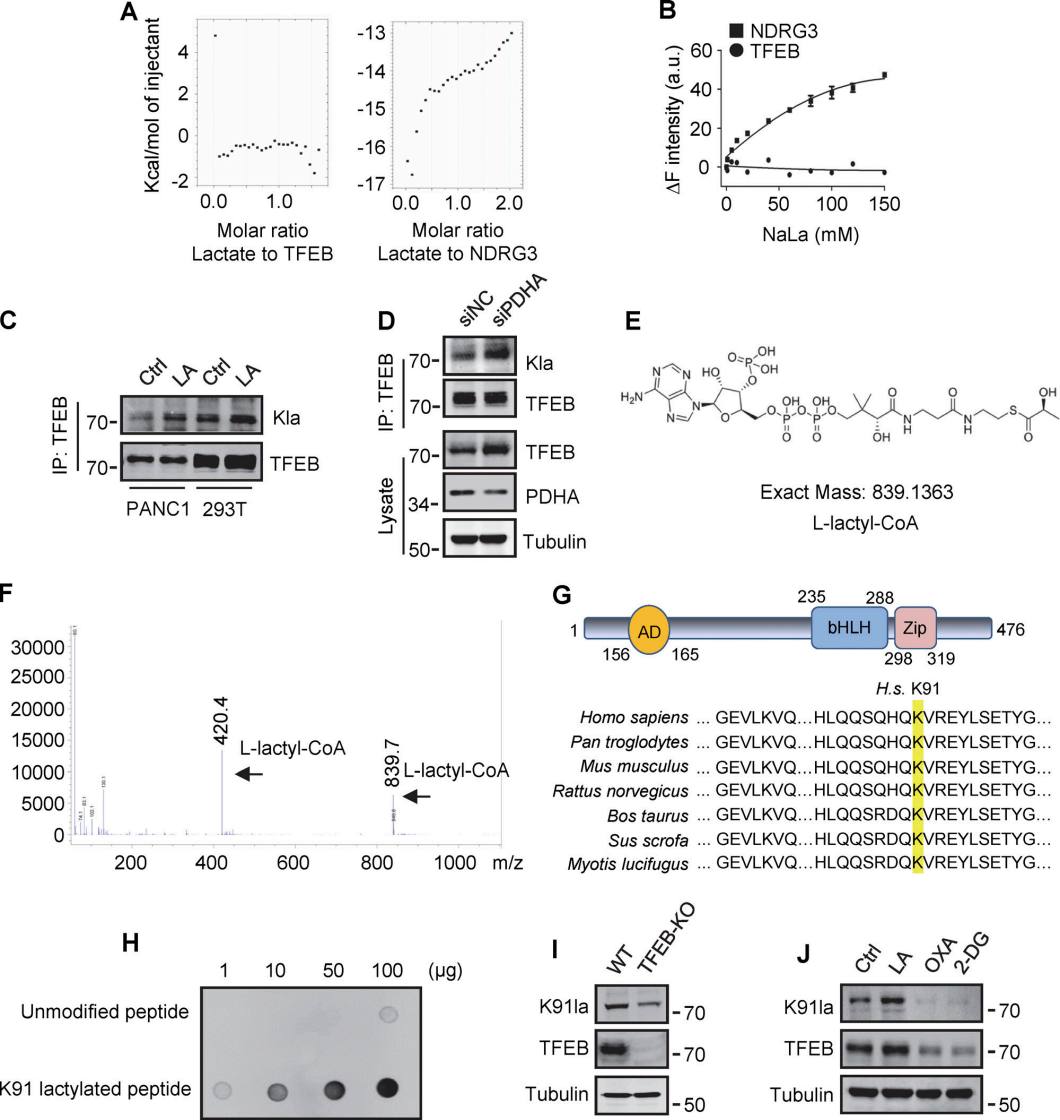
**Lactylation of TFEB at K91. (A)** The interaction of lactate and TFEB or NDRG3 determined by isothermal titration calorimetry. 0.5 mM purified TFEB or NDRG3 was used. **(B)** The binding affinity of lactate to purified TFEB or NDRG3 determined by tryptophan fluorescence quenching assay. **(C)** Lactylation of TFEB in PANC1 cells and HEK293T cells after lactic acid (LA) treatment. Immunoprecipitated TFEB was blotted with anti-Kla. **(D)** Lactylation and expression of TFEB in HeLa cells cultured with PDHA siRNA for 72 h. Immunoprecipitation (IP) with anti-TFEB and western blotting using anti-Kla were performed. **(E)** Structure of L-lactyl-CoA. **(F)** MS verification of synthesized L-lactyl-CoA. **(G)** Structure diagram of TFEB protein domain and alignment of the TFEB amino acid sequence across different mammalian species. AD, activation domain; bHLH, basic helix-loop-helix domain; Zip, zipper domain. **(H)** Dot blot assay confirming the specificity of the antibody targeting TFEB lactyl-K91. Unmodified and lactyl-K91 peptides were dripped onto nitrocellulose membranes and probed with K91la antibody. **(I and J)** TFEB lactylation at K91 in TFEB-KO HeLa cells (I) or HeLa cells subjected to lactic acid, OXA, or 2-DG treatment for 24 h (J). Protein blotting used the specific antibody against lactyl-K91 of TFEB. All molecular weights are in kD. Source data are available for this figure: SourceData FS4.

**Figure 5.**
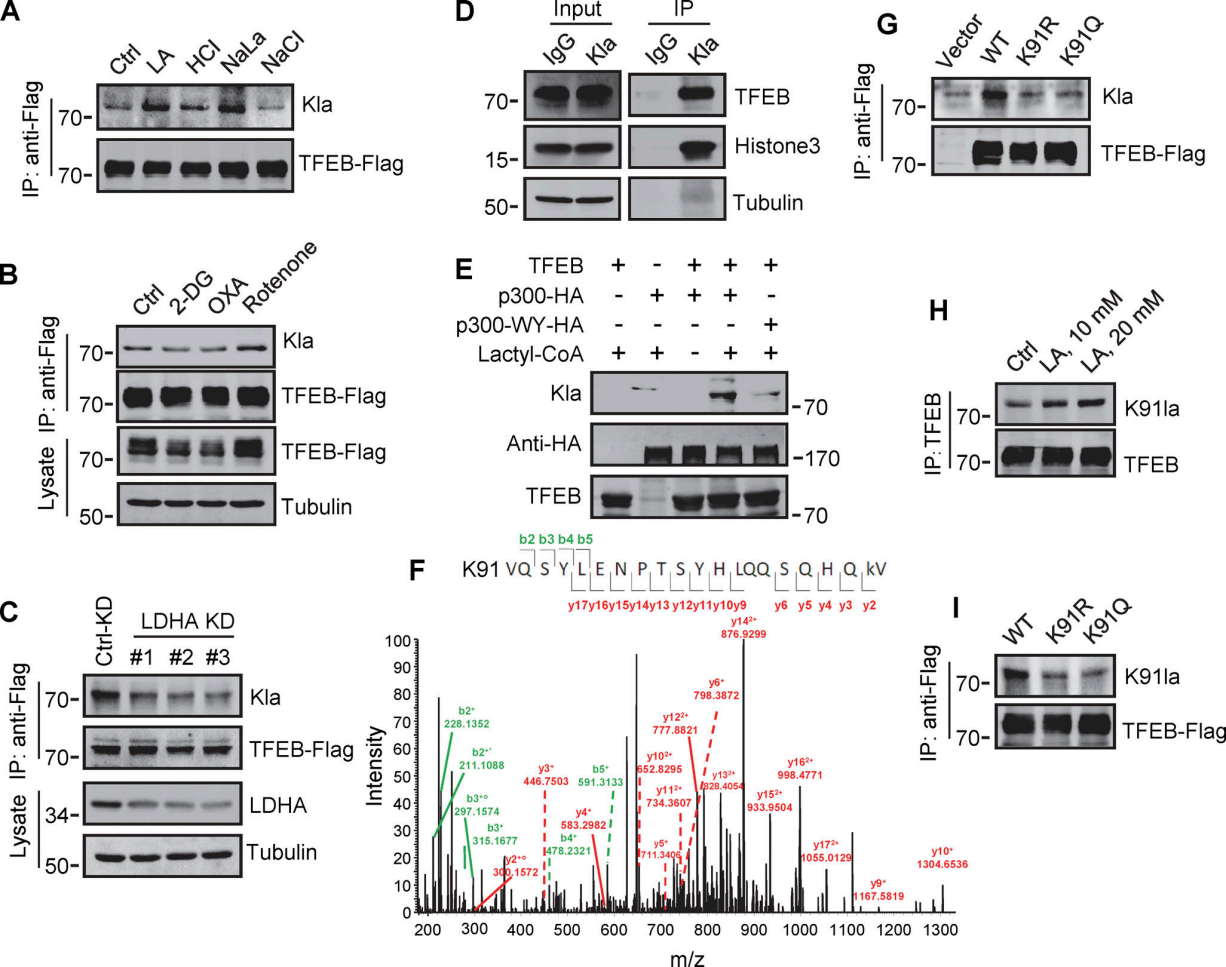
**Identification of K91 lactylation of TFEB. (A–C)** Lactylation analysis of TFEB-Flag in cells. Transient TFEB-Flag expressing HeLa cells were treated with the indicated chemicals (A and B) or cultured with three LDHA siRNAs for 48 h followed by transfection with TFEB-Flag for 24 h (C). TFEB-Flag immunoprecipitated by anti-Flag was blotted with anti-Kla. **(D)** Lactylation of endogenous TFEB. Lactylated proteins in lactic acid (LA)–treated HeLa cells were immunoprecipitated (IP) by anti-Kla beads and analyzed by western blotting using anti-TFEB and anti-histone3. IgG was used as a control for anti-Kla antibody. **(E)** In vitro TFEB lactylation assay. Purified TFEB was incubated with HA-tagged p300 immunoprecipitated from HEK293T cells in the presence or absence of lactyl-CoA. TFEB lactylation was analyzed by western blotting using anti-Kla. **(F)** Mass spectrometric analysis of lactylation site in TFEB immunopurified from lactic acid–treated HeLa cells. **(G)** Lactylation of TFEB-K91R and TFEB-K91Q in HeLa cells 24 h after transfection. K91R: Lys 91 was replaced by Arg. K91Q: Lys 91 replaced by Gln. **(H)** Lactylation of TFEB at K91 in HeLa cells after lactic acid treatment. TFEB was immunoprecipitated using anti-TFEB and blotted with an antibody specifically against lactyl-K91 (K91la). **(I)** K91 lactylation of TFEB-K91R and TFEB-K91Q. All molecular weights are in kD. Source data are available for this figure: SourceData F5.

To explore the mechanism by which lactate inhibits TFEB–WWP2 interactions and thus TFEB degradation, we tested whether lactate directly interacts with TFEB as it does with NDRG3 (Lee et al., 2015) and MAVS (Zhang et al., 2019b). Recombinant TFEB protein was purified and its binding affinity to lactate was determined by in vitro isothermal titration assay with purified NDRG3 as control. Interestingly, although NDRG3 titrated by lactate showed increased heat production, no significant heat release was observed in TFEB titrated by lactate under the same conditions (Fig. S4 A). In addition, the tryptophan quenching experiment showed that the addition of sodium lactate resulted in the fixed tryptophan fluorescence quenching of purified NDRG3, but had no effect on the intrinsic tryptophan fluorescence of purified TFEB (Fig. S4 B). These results suggested that lactate may not directly interact with TFEB through non-covalent binding. Therefore, we used a specific antibody against lactyl-lysine (Kla) to test whether lactate triggers the lactylation of TFEB. Clearly, lactate or sodium lactate treatment enhanced the lactylation modification of TFEB-Flag transfected in HeLa cells and the lactylation of endogenous TFEB in PANC1 cells and HEK293T cells (Fig. 5 A and Fig. S4 C). Consistently, rotenone treatment promoted TFEB lactylation, while 2-DG or OXA treatment or LDHA KD reduced TFEB lactylation (Fig. 5, B and C). In addition, anti-Kla beads pulled down large amounts of endogenous TFEB along with histone3 from the lysates of lactate-treated cells (Fig. 5 D), knocking down the key component of the pyruvate dehydrogenase complex (PDH) also increased levels of TFEB lactylation and TFEB protein (Fig. S4 D). Finally, we conducted in vitro lactylation assays by incubating purified recombinant TFEB with histone acetyltransferase p300 immunoprecipitated from cells, which has previously been shown to mediate histone lactylation (Zhang et al., 2019a). In the presence of synthetic L-lactyl-CoA identified by MS (Fig. S4, E and F), lactylation was observed in TFEB incubated with p300-HA instead of transferase-inactivated p300-WY-HA (Bordoli et al., 2001) (Fig. 5 E). Taken together, these data suggest that TFEB can undergo lactylation, and that an increase in lactic acid in cells promotes this modification of TFEB.

To determine the lactylation site on TFEB, we performed MS of immunopurified TFEB protein from lactate-treated cells. K91, located on the N-terminus of TFEB and conserved in mammalian species (Fig. S4 G), appeared as a potential residue (Fig. 5 F). We then created a TFEB mutant in which K91 was replaced by arginine or glutamine. Using pan-anti-Kla antibodies, we found that TFEB-K91R and TFEB-K91Q were almost completely non-lactylated in cells (Fig. 5 G). In addition, by developing and using a specific anti-lactyl-K91 antibody (Fig. S4, H and I), we showed that lactate treatment promoted the lactylation of TFEB at K91, while 2-DG or OXA treatment substantially weakened it (Fig. 5 H and Fig. S4 J), supporting K91 as the main lactylation site of TFEB (Fig. 5 I).

### Lactylation inhibits TFEB–WWP2 interaction and TFEB degradation

**Figure 6.**
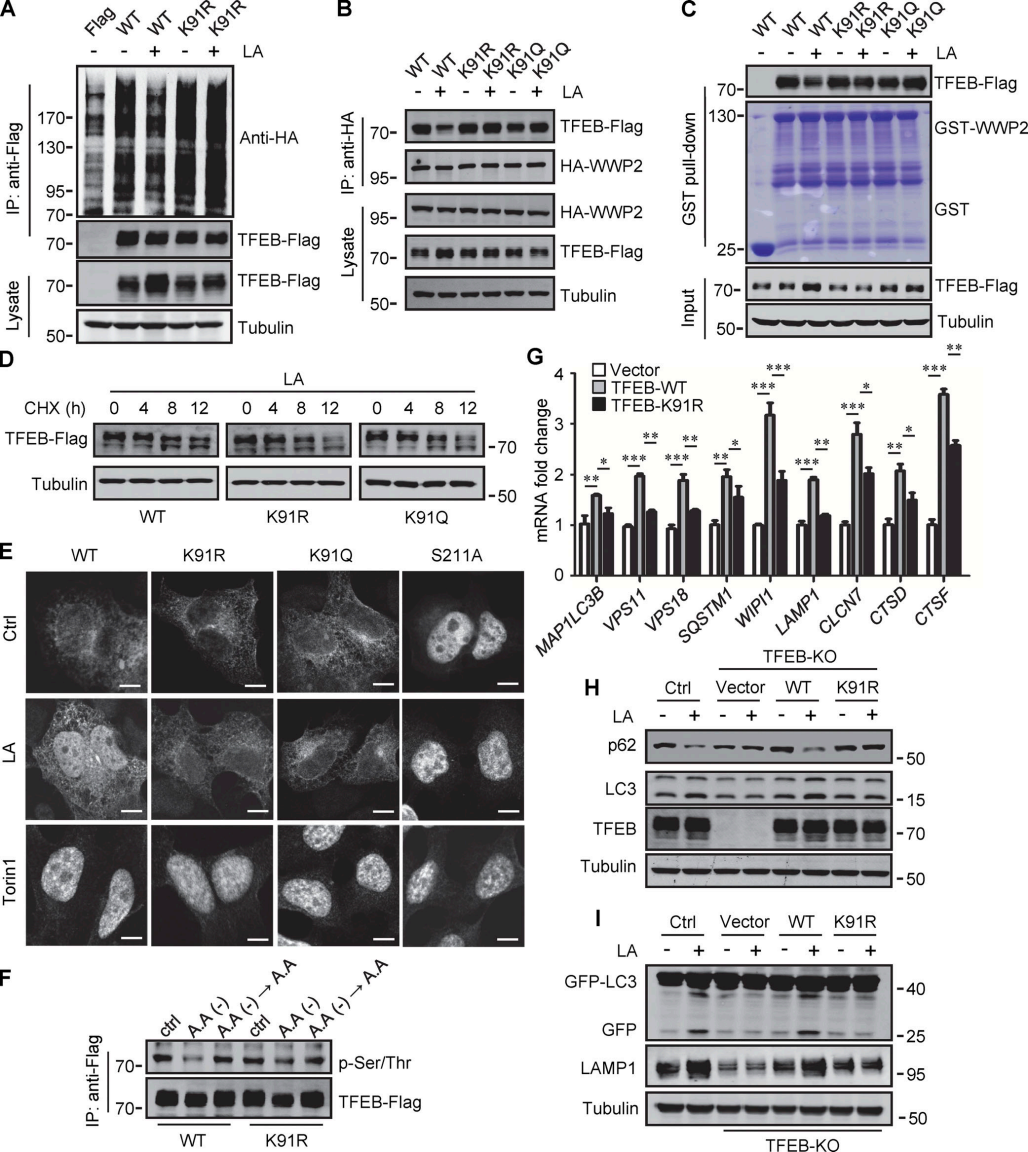
**Lactylation prevents TFEB–WWP2 interaction and TFEB degradation. (A)** Ubiquitination of TFEB-K91R in cells. HeLa cells expressing HA-ubiquitin were transfected with the indicated plasmids, then were treated with or without lactic acid (LA). **(B)** Co-immunoprecipitation (IP) of Flag-tagged TFEB-K91R and TFEB-K91Q with HA-WWP2 in co-transfected HeLa cells. Cells were treated with or without lactic acid 24 h after transfection. **(C)** In vitro pull-down assay of WWP2–TFEB binding. Purified GST-WWP2 beads were incubated with lysates of Flag-tagged TFEB-expressing HEK293 cells treated with or without lactic acid. GST-WWP2 bound TFEB-Flag was detected by western blotting using anti-Flag. **(D)** Degradation of TFEB-Flag in HeLa cells. Flag-tagged TFEB-WT or TFEB mutants were treated with lactic acid in the presence of CHX for the indicated time, then the proteins were detected by western blot using anti-Flag. **(E)** Distribution of TFEB. HEK293 cells transiently expressing Flag-tagged TFEB were treated with lactic acid or Torin1 and stained with anti-Flag. Scale bars, 5 μm. **(F)** Phosphorylation of TFEB. HEK293T cells transiently expressing TFEB-Flag or TFEB-K91R-Flag were cultured in amino acid-free medium for 4 h. After amino acid supplementation for 1 h, TFEB were immunoprecipitated and blotted with an antibody against phosphorylated serine/threonine. **(G)** Expression of TFEB target gene mRNA in HeLa cells expressing TFEB-Flag or TFEB-K91R-Flag. The Data are from three independent experiments and presented as mean ± SEM; *P < 0.05, **P < 0.01, ***P < 0.001. **(H)** Levels of p62 and LC3 in TFEB-KO HeLa cells transfected with TFEB-WT or TFEB-K91R. **(I)** Free GFP production and LAMP1 expression in TFEB-KO HeLa cells transfected with TFEB-WT or TFEB-K91R and GFP-LC3. Cells were treated with or without lactic acid. All molecular weights are in kD. Source data are available for this figure: SourceData F6.

To verify that K91 lactylation plays a role in lactate-induced TFEB stabilization, we first examined its effect on TFEB ubiquitination. When expressed in cells, the basal ubiquitination of TFEB-K91R was slightly higher than that of WT-TFEB, and lactic acid treatment neither reduced the ubiquitination of TFEB-K91R nor caused significant accumulation of TFEB-K91R (Fig. 6 A). Immunoprecipitation and in vitro pull-down analysis using purified recombinant GST-WWP2 showed that the interaction of TFEB-K91R and TFEB-K91Q with WWP2 could not be weakened by lactate treatment (Fig. 6, B and C). We then compared the degradation of WT-TFEB, TFEB-K91R, and TFEB-K91Q in lactic acid–treated cells in the presence of CHX and found that the mutation at K91 accelerated the degradation of TFEB (Fig. 6 D). These results suggest that the lactylation at K91 can inhibit the ubiquitination and degradation of TFEB by disrupting the interaction between TFEB and WWP2.

**Figure S5.**
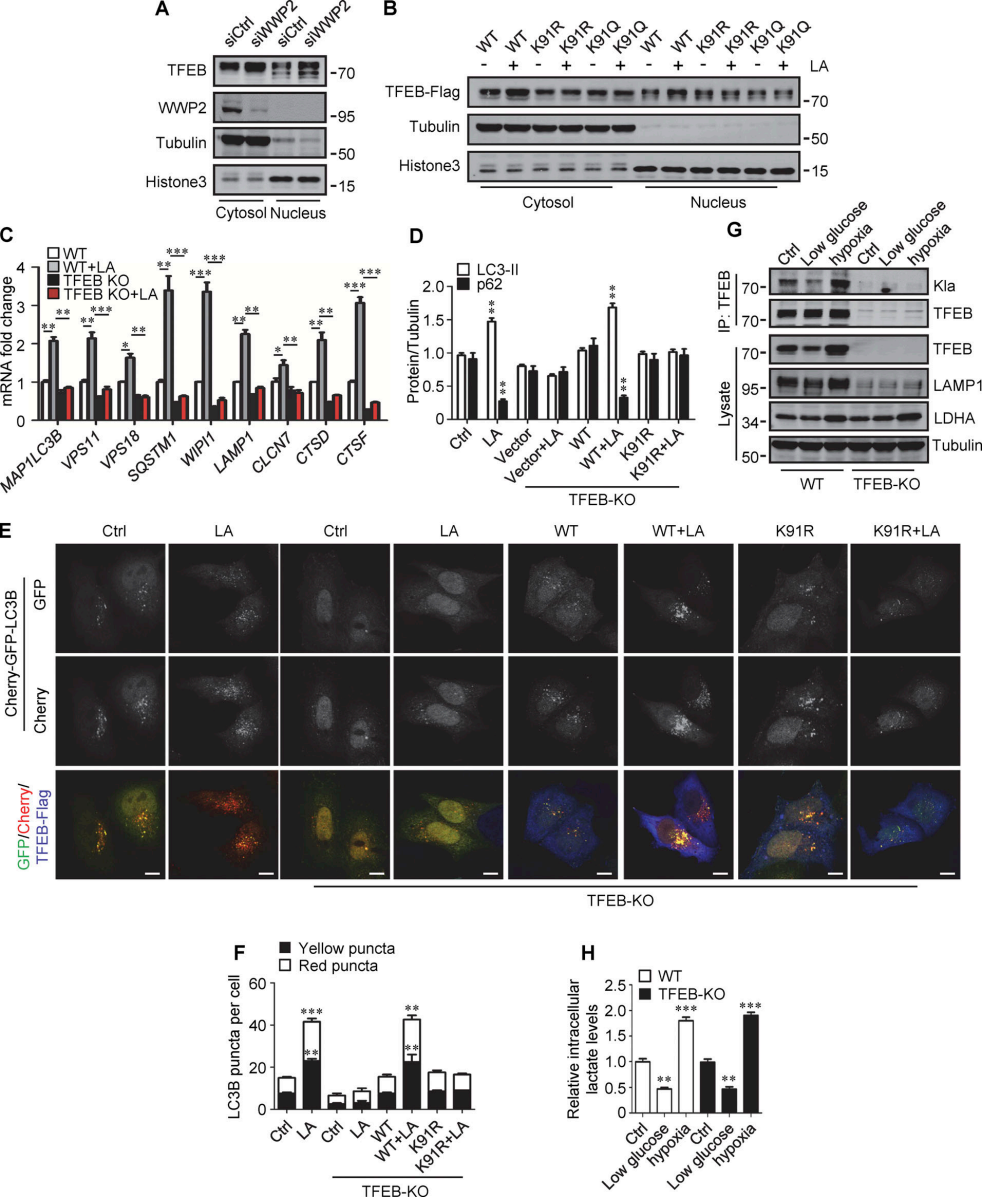
**Lactylation prevents TFEB–WWP2 interaction and TFEB degradation. (A)** Levels of TFEB and WWP2 in the cytoplasmic and nuclear fractions of HeLa cells treated with control or WWP2 siRNA for 72 h. **(B)** TFEB-Flag expression in cytoplasmic and nuclear fractions of HeLa cells with or without lactic acid (LA) treatment. Tubulin and histone3 were used as cytoplasmic and nuclear markers, respectively. **(C)** mRNA levels of TFEB target genes in WT and TFEB-KO HeLa cells with or without lactic acid treatment. Data are presented as mean ± SEM (*n* = 3). **(D)** Quantification of p62 and LC3-II protein levels in Fig. 6 H. Data are presented as mean ± SEM (*n* = 3). **(E)** Assessment of autophagosomes and autolysosomes in HeLa cells using Cherry-GFP-LC3B. TFEB-KO cells transfected with Cherry-GFP-LC3B, or Cherry-GFP-LC3B, and TFEB-WT or TFEB-K91R, were treated with or without lactic acid; scale bars, 10 μm. **(F)** Statistical analysis of LC3 puncta count per cell in E. Data are from 30 cells of three independent experiments and presented as mean ± SEM. **(G)** TFEB lactylation in WT and TFEB-KO HeLa cells cultured with 5 mM glucose or 1% oxygen for 24 h. Immunoprecipitated (IP) TFEB was blotted with anti-Kla. Note the expression of TFEB, LAMP1, and LDHA in cell lysates. **(H)** The intracellular lactate levels measured in HeLa cells treated as described in G. Data are presented as mean ± SEM (*n* = 3). *P < 0.05, **P < 0.01, ***P < 0.001. All molecular weights are in kD. Source data are available for this figure: SourceData FS5.

We further investigated the effect of lactylation on the intracellular localization of TFEB. Phosphorylation at S211 by mTORC1 traps TFEB in the cytoplasm and TFEB-S211A mutant is primarily distributed in the nucleus (Roczniak-Ferguson et al., 2012). Using TFEB-S211A as a control, we found that lactic acid treatment increased the nuclear distribution of WT-TFEB, but not TFEB-K91R and TFEB-K91Q, whose nuclear translocations were strongly stimulated by the mTORC1 inhibitor Torin1 (Fig. 6 E). Consistent with this, the phosphorylation level of TFEB-K91R in cells was similar to WT-TFEB and exhibited the same response to amino acid depletion and supplementation as WT-TFEB (Fig. 6 F). We also used subcellular fractionation to quantify the levels of TFEB in the cytoplasm and nucleus. As with lactic acid treatment, WWP2 KD increased TFEB in the cytoplasm and nucleus (Fig. S5 A). However, unlike WT-TFEB, lactic acid did not increase the amount of TFEB-K91R and TFEB-K91Q in the nucleus (Fig. S5 B). Taken together, these results suggest that the lactylation of TFEB is not directly related to the phosphorylation of TFEB. The increase in nuclear TFEB in lactate-treated cells is attributed to the inhibition of lactation-mediated TFEB degradation.

We then evaluated the role of TFEB and its lactylation in lactate-activated autophagy and lysosomal biogenesis. Using TFEB-KO (knockout) HeLa cells generated by CRISPR/Cas9 system, we revealed that the loss of TFEB eliminated the stimulating effect of lactate on mRNA expression of autophagy- and lysosome biogenesis-related genes (Fig. S5 C). When the reintroduction of WT-TFEB was able to restore their expression, the introduction of TFEB-K91R only partially rescued them (Fig. 6 G). Compared with WT cells, lactic acid failed to trigger LC3-II production and p62 reduction in TFEB-KO cells, which were recovered by WT-TFEB but not TFEB-K91R transfection (Fig. 6 H and Fig. S5 D). Taking advantage of the resistance of GFP to cathepsin-mediated digestion (Mizushima et al., 2010), we measured autophagy flux by checking GFP-LC3 processing in lysosomes. In HeLa cells expressing GFP-LC3, lactic acid treatment led to the production of free GFP (Fig. 6 I). Loss of TFEB inhibited free GFP production, which was retrieved by reintroduction of WT-TFEB rather than TFEB-K91R (Fig. 6 I). Based on the different sensitivities of GFP and Cherry to lysosomal acidic environments, we further used the Cherry-GFP-LC3 reporter to detect autophagy flux (Klionsky et al., 2021). Apparently, lactic acid treatment increased the number of both GFP^+^/Cherry^+^ puncta (autophagosomes) and GFP^−^/Cherry^+^ (autolysosomes) in WT but not TFEB-KO cells (Fig. S5, E and F), and re-expression of WT-TFEB instead of TFEB-K91R in TFEB-KO cells restored the production of the puncta (Fig. S5, E and F). These results indicate that TFEB lactylation plays a crucial role in mediating increased autophagy flux stimulated by lactate.

Finally, we cultured cells with low glucose or hypoxia to verify that TFEB lactylation can be regulated under physiological conditions. Obviously, both TFEB lactation and TFEB protein levels were reduced in cells cultured with low glucose that produced less lactic acid (Fig. S5, G and H). On the contrary, under hypoxia conditions that promote lactate production, the lactylation and expression of TFEB in cells were increased (Fig. S5, G and H).

### TFEB lactylation in pancreatic cancer

**Figure 7.**
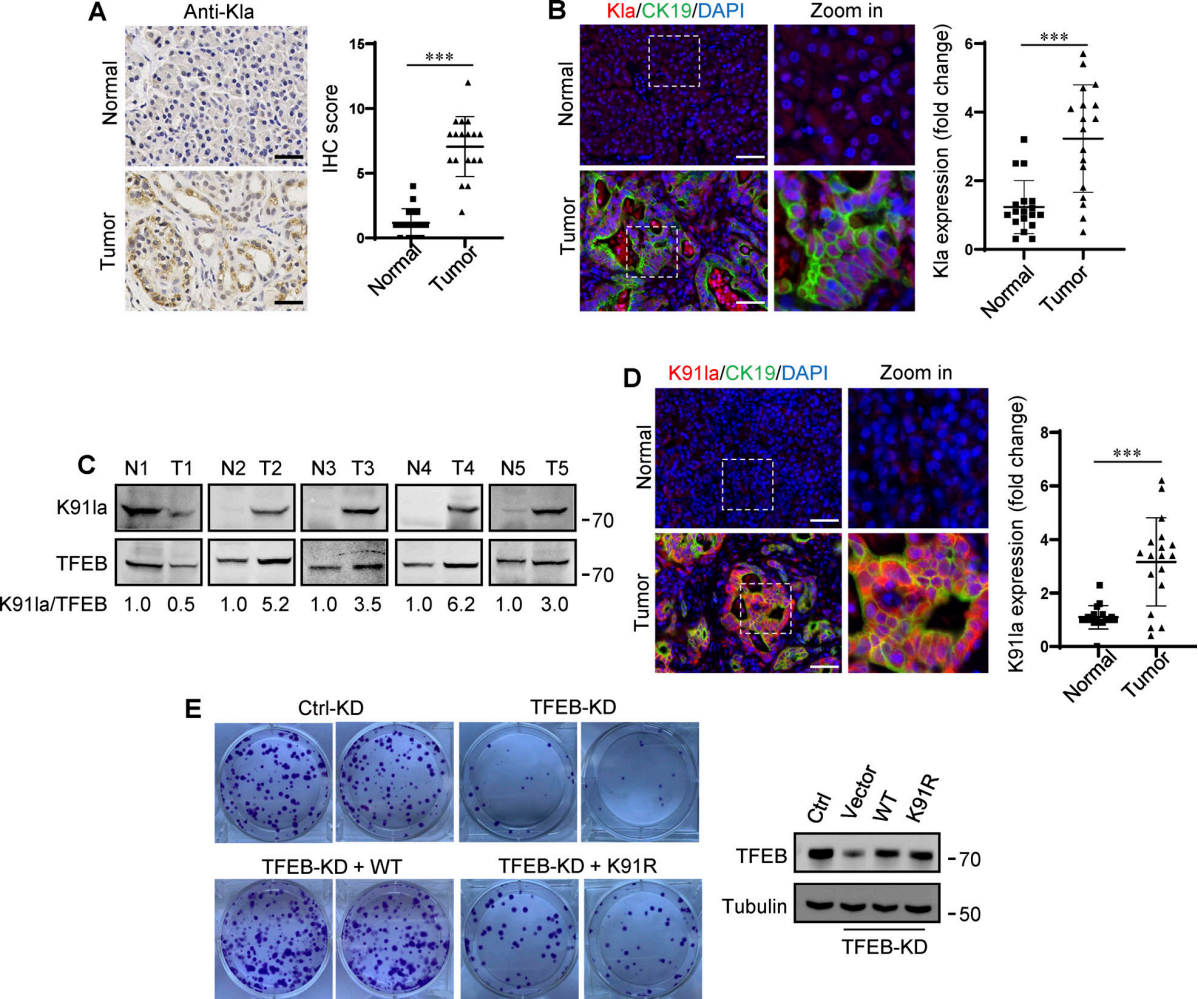
**Upregulation of TFEB lactylation in pancreatic cancer. (A)** Immunohistochemical (IHC) images of representative PDA (*n* = 18) and adjacent normal (*n* = 18) samples stained with anti-Kla. Scale bars, 10 μm. **(B)** Representative immunofluorescent images of PDA and normal tissues stained with anti-Kla and anti-CK19, and DAPI. Scale bars, 10 μm. **(C)** Lactylation of TFEB K91 in five pairs of PDA (T) and matched adjacent normal tissues (N). The specific K91la antibody was used for western blotting and the relative intensity ratios of the lactylated TFEB K91 band to the corresponding TFEB band are shown. **(D)** Representative immunofluorescent images of PDA and normal samples stained with anti-TFEB-K91la and anti-CK19. Scale bars, 10 μm. **(E)** In vitro colony-forming ability of PANC1 cells. Cells were transfected with Flag-tagged TFEB-WT or TFEB-K91R after TFEB RNAi and were stained with crystal violet 2 wk later. The expression of TFEB protein in these cells is shown. The quantitative data show relative whole-section expression of lactylated proteins (*n* = 18 samples) (A and B) and lactylated TFEB (*n* = 18 samples) (D). The data are presented as mean ± SEM. ***P < 0.001. All molecular weights are in kD. Source data are available for this figure: SourceData F7.

In human tumors, particularly PDA and non-small cell lung cancer, elevated TFEB expression is associated with tumor progression (Giatromanolaki et al., 2015; Perera et al., 2015). To understand whether rapidly proliferating cells contain high lactylated TFEB, we first performed immunohistochemistry using the pan-anti-Kla antibody to detect protein lactylation in tissues of PDA patients after clinical surgery. We found that human PDA samples showed strong protein lysine lactylation staining in both the cytoplasm and nucleus compared with the matched normal control tissues with almost undetectable lactylation signals (Fig. 7 A). Costaining with cytokeratin 19 (CK19), a marker of tumor epithelia, confirmed lactylation in the nucleus of PDA cancer cells (Fig. 7 B). Importantly, using the specific antibody targeting lactyl-K91 in TFEB, western blotting showed a marked increase in TFEB lactylation in most tested PDA samples (Fig. 7 C). Costaining of tissue samples with anti-lactyl-K91 and anti-CK19 antibodies validated that TFEB lactylation does indeed occur in PDA cancer cells (Fig. 7 D). Finally, we examined the effect of TFEB lactylation on PANC1 cell proliferation by knocking down TFEB and re-expressing WT-TFEB or TFEB-K91R. Clearly, KD of TFEB reduced the clonal growth of PANC1 cells, and reintroduction of WT-TFEB completely restored the growth, while reintroduction of WT-TFEB only slightly mitigated the decrease (Fig. 7 E).

## Discussion

In this study, we found a link between intracellular lactate and the key autophagy transcription factor TFEB. We demonstrate that intracellular lactate promotes autophagy and lysosomal biogenesis by inducing lactylation-dependent stabilization of TFEB. In addition to identifying a novel posttranslational modification of TFEB, our findings suggest that this lactylation-mediated quality control mechanism of TFEB is associated with high autophagy in rapidly proliferating cells such as cancer cells, where glucose metabolism is reprogrammed and high lactate is produced (Bronietzki et al., 2015; Jacquin and Apetoh, 2018).

The influence of lactic acid on autophagosome formation and lysosomal biogenesis has led us to explore the link between lactate and TFEB. Previously, TFEB expression was found to be upregulated in cancer cells, but the mechanism was not clear. Although the increase in importin expression may promote TFEB nuclear transport, thereby stabilizing TFEB (Perera et al., 2015), our results suggest another mechanism by which TFEB accumulates in transformed cells. We provide evidence that lactylation inhibits TFEB degradation and human cancers have high levels of TFEB lactylation. Therefore, lactylation-mediated TFEB stabilization may contribute to high TFEB levels in cancer cells. Although TFEB mRNA levels are also elevated (Perera et al., 2015), given that they are only enhanced in cells stimulated by lactate for a long time and that there is a positive feedback on TFEB transcription, we suggest that inhibition of TFEB degradation by lactate is the primary trigger.

Histone lactylation is associated with many cellular processes by promoting the expression of multiple genes (Hagihara et al., 2021; Pan et al., 2022). Recently, chromatin-associated high mobility group box-1 (HMGB1) and methyltransferase-like 3 (METTL3) have been found to undergo lactylation. Lactylation promotes the release of HMGB1 from macrophages and enhances the interaction between METTL3 and target RNA (Gu et al., 2022; Yang et al., 2022). We demonstrate here that transcription factors can become targets of lactylation. Interestingly, lactylation does not alter the subcellular localization of TFEB nor does it interfere with mTOR’s inhibition of TFEB nuclear translocation. While phosphorylation and acetylation regulate TFEB activity by affecting TFEB nuclear transport and binding of TFEB to its target gene promoter (Wang et al., 2020), lactylation primarily controls TFEB proteasome degradation. Surprisingly, our results exclude the known TFEB ubiquitin E3 ligase STUB1 from being involved in the lactate-regulated TFEB degradation, although STUB1 can indeed interact with TFEB. Instead, we found that WWP2 is a novel TFEB E3 ubiquitin ligase. By using TFEB-unbound and inactivated WWP2 mutants, we clearly showed that WWP2 interacts with TFEB and mediates TFEB ubiquitination and proteasomal degradation in vitro and in cells. Intriguingly, we revealed the interaction between TFEB and E3 ubiquitin ligase RNF114. However, overexpression of RNF114 did not lead to the degradation of TFEB, indicating that the interaction may be involved in other TFEB-related events rather than mediating TFEB turnover. More importantly, we determined that lactylation reduces TFEB degradation by hindering the interaction between TFEB and WWP2. Since DNA binding of MiT/TEF factors requires them to form homodimers or heterodimers, and acetylation affects the dimerization of TFEB in the nucleus (Wang et al., 2020), it would be interesting to know whether lactylation has a similar effect.

Acetyl-transferase p300 can catalyze the transfer of lactyl group from lactyl-CoA to histones and HMGB1 (Yang et al., 2022; Zhang et al., 2019a), while histone deacetylase HDAC 1–3 acts as a histone delactylase (Moreno-Yruela et al., 2022). We found that p300 catalyzes TFEB lactylation in vitro, but its role in cells remains to be verified. We previously found that mTORC1 phosphorylates and activates p300 (Wan et al., 2017). This indicates that mTORC1 may positively regulate TFEB lactylation and stability through p300. Given that TFEB promotes the recruitment of mTORC1 to lysosomes through transcriptional activation of RagD (Di Malta et al., 2017), these data suggest a complex mutual regulation between mTORC1 and TFEB, particularly in cancer cells. This interaction between mTORC1 and TFEB may contribute to sustained high levels of synthetic and catabolic metabolism in cancer cells. In addition to mTORC1, the localization and activity of TFEB are also regulated by other kinases (Ferron et al., 2013; Martina et al., 2016; Settembre et al., 2011) and phosphatases (Martina and Puertollano, 2018; Medina et al., 2015). The changes in phosphorylation of these kinase or phosphatase target sites may also provide mechanisms for activating TFEB without inhibiting mTORC1, although it is not yet clear whether the activity of these kinases or phosphates is affected by lactate. Nonetheless, our results support the fact that overexpression of TFEB can sufficiently enhance autophagy gene expression, autophagosome formation, and autophagic degradation (Settembre et al., 2011, 2013).

Due to the large number of disordered regions, the structure of the full-length TFEB has not yet been determined. Our MS analysis identified K91 as the only lactylation site. When lactylation-induced conformational changes in TFEB are certainly a cause for consideration, K91 is structurally distant from the C-terminal PPGY motif according to AlphaFold. How the lactylation at K91 interferes with the binding of WWP2 remains to be studied, and one possibility is that other proteins are involved in the interaction between TFEB and WWP2.

### Limitations of the study

p300 catalyzes TFEB lactylation in vitro, but it is not yet clear whether it plays a role in cells, and other acetyltransferases may also be involved. We found that TFE3 can undergo lactylation like TFEB, but the lactylation site on TFE3 is a mystery as there is no corresponding K91 site in TFE3. Furthermore, determining the degree to which endogenous TFEB is modified by lactate remains challenging.

## Materials and methods

### Cell culture and transfection

HEK293, HEK293T, HeLa, and PANC1 cells were cultured in Dulbecco’s modified Eagle medium (DMEM; Gibco) supplemented with 10% fetal bovine serum (FBS) and maintained in a 37°C incubator with a humidified, 5% CO_2_ atmosphere. Transient transfection of plasmids was performed using Lipofectamine 2000 (Invitrogen) according to the manufacturer’s instructions. Cells were analyzed 18–24 h after transfection.

### Antibodies and reagents

Antibodies to TFEB (4240, 37785), TFE3 (14779), histone 3 (4499), STUB1 (2080), AKT (9272), phospho-AKT1 (9271), S6K1 (9202), phospho-S6K1 (9205), COX IV (4850), and TOM20 (42406) were purchased from Cell Signaling Technology; antibodies to His (sc-803), MCT1 (sc-365501), MCT4 (sc-376140), and LAMP1 (sc-17768) were purchased from Santa Cruz; antibodies to p62 (18420), LDHA (19987), PDHA (18068), and ubiquitin (10201) were purchased from Proteintech; antibodies to α-Tubulin (M175-3), Myc (M192-3S), Flag (M185-3B), HA (M180-3s), GFP (M048-3), and GST (M071) were purchased from MBL; antibody to LC3 was purchased from Sigma-Aldrich; antibodies to WWP2 (ab103527), EGFR (ab32562), CK19 (ab76539), and PGC1α (ab106814) were purchased from Abcam; antibody to *L*-lactyl lysine (PTM-1401) was purchased from PTM Biolabs.

L-lactic acid (L1750), sodium *L*-lactate (L7022), sodium oxamate (O2751), 2-Deoxy-D-glucose (D8357), rotenone (R8875), chloroquine (C6628), CHX (C7698), MG132 (SML1135), and LLOMe (L7393) were purchased from Sigma-Aldrich; Torin1 (4247) was purchased from Tocris Biosciences; EGF (P7109) was purchased from MCE; and lactate colorimetric assay kit II (K627-100) was purchased from Biovision.

Unless otherwise stated, the chemicals were used as follows: *L*-lactic acid and sodium *L*-lactate, 20 mM, 24 h; HCl, 0.3 μM, 24 h; sodium oxamate, 20 mM, 24 h; 2-Deoxy-D-glucose, 10 mM, 24 h; rotenone, 10 nM, 24 h; torin1, 250 nM, 4 h; chloroquine, 50 μM, 6 h; bafilomycin A1, 100 nM, 6 h; MG132, 5 μM, 6 h; CHX, 5 μM; LLOMe, 1 mM, 30 min. Cells cultured in amino acid–free medium containing 10% dialyzed FBS for 4 h were referred to as amino acid starvation.

### Western blot and immunoprecipitation

For western blot, whole cell lysates were prepared in radioimmunoprecipitation assay buffer (50 mM Tris-HCl, pH 7.4, 150 mM NaCl, 1% sodium deoxycholate, 0.1% SDS, 1 mM EDTA) supplemented with complete protease inhibitor cocktail. Cells were incubated for 20 min at 4°C then spun at 15,000 rpm for 15 min at 4°C. The supernatant was used to determine the total protein concentration using the BCA Protein Assay Kit (23225; Thermo Fisher Scientific). Protein lysates were denatured and separated by SDS-PAGE and then transferred to a polyvinylidene difluoride membrane. After blocking with 5% (wt/vol) bovine serum albumin for 1 h at room temperature, the membrane was hybridized with corresponding primary antibodies and secondary antibodies. Specific bands were captured utilizing an Odyssey infrared imaging system (LI-COR Biosciences). Protein bands were quantified using Quantity One software. The signals from the protein of interest were normalized with that of the loading control.

For immunoprecipitation, cells were lysed in NP-40 lysis buffer (20 mM Tris-HCl, pH 7.5, 0.5% NP-40, 1 mM MgCl_2_, 150 mM NaCl, 1 Mm CaCl_2_, 10% glycerol, 1 mM Na_4_P_2_O_7_) supplemented with cocktail. Cells were incubated for 20 min at 4°C then spun at 15,000 rpm for 15 min at 4°C. The supernatant was incubated on Anti-Flag-tag mAB-magnetic beads (B26102; Selleck), anti-HA -tag mAB-magnetic beads (B26202; Selleck), and anti-Kla-tag mAB-beads (PTM-1404; PTM Biolabs) at 4°C for 3 h. For endogenous immunoprecipitationss, the supernatant was incubated with 2 μl of TFEB antibody (37785) or WWP2 antibody (ab103527) for overnight at 4°C, followed by the addition of protein A agarose beads (BD0047; Bioworld) for 3 h. Then the beads were washed five times using lysis buffer and subjected to western blot.

### Stable cell line construction

TFEB-KO HeLa cells were generated in our laboratory using the CRISPR/Cas9 system as described previously (Wang et al., 2020). The design of CRISPR single guide RNAs (sgRNAs) was aided by publicly available software provided by MIT (Cong et al., 2013). sgRNA oligos for TFEB is 5′-AGT​ACC​TGT​CCG​AGA​CCT​AT-3′. Primers were annealed using the following conditions: 98°C for 5 min, 80°C for 5 min, 70°C for 5 min, 68°C for 5 min, and 50°C for 5 min. The annealed primers were inserted into the linearized sgRNA vector pEP-KO Z1779 plasmid containing expression cassettes of WT Cas9 and puromycin-resistant gene using SapI sites. TFEB-KO HeLa cells were created by transient transfection of pEP-TFEB-KO plasmid using Lipofectamine 2000 followed by selection with puromycin (2.5 μg/ml). The KO efficiency of single-cell clone was examined by western blot (Wang et al., 2020). Myc-LDHA plasmid was generated by cloning human LDHA DNA fragments into pQCXIP. HeLa cells stably expressing Myc-LDHA were created by transfection with pQCXIP-LDHA for 72 h and selected by treatment with puromycin. Cells were serially diluted into 96-well plates to select for single colony clones.

### EGFR degradation assay

After being cultured in serum-free DMEM for 12 h, HeLa cells with or without stably expressing Myc-LDHA were treated with EGF (200 ng/ml) in serum-free medium on ice for 15 min. Then, cells were washed with PBS to remove EGF and were recultured in a serum-free medium at 37°C. At indicated time points, the cells were lysed and subjected to western blot with EGFR antibody.

### RNA extraction and real-time PCR

Total RNA from cells was extracted with TRIzol Reagent (Invitrogen). Reverse transcription was performed using First Strand cDNA Synthesis Kit (TOYOBO). Real-time quantitative PCR (qPCR) was performed with SYBR Premix EX Taq (TaKaRa) on the CFX96 Touch Real-Time Detection System (Bio-Rad Laboratories). Relative expressions of specific genes were calculated by the 2^−DDCt^ method normalized against β-Actin. The primers were used in this study, *TFEB* forward, 5′-GGC​AAC​AGT​GCT​CCC​AAT​AGC-3′, *TFEB* reverse, 5′-CCC​AAC​TCC​TTG​ATG​CG GTCA-3′; *MAP1LC3B* forward, 5′-ACC​ATG​CCG​TCG​GAG​AAG-3′, *MAP1LC3B* reverse, 5′-ATC​GTT​CTA​TTA​TCA​CCG​GGA​TTT​T-3′; *VPS11* forward, 5′-CAA​GCC​TAC​AAA​CTA​CGG​GTG-3′, *VPS11* reverse, 5′-GAG​TGC​AGA​GTG​GAT​TGC​CA-3′; *VPS18* forward, 5′-CAC​TCG​GGG​TAT​GTG​AAT​GCC-3′, *VPS18* reverse, 5′-TCG​GAA​GGG​GTG​AAG​TCA​ATG-3′; *SQSTM1* forward, 5′-ATC​GGA​GGA​TCC​GAG​TGT-3′, *SQSTM1* reverse, 5′-TGG​CTG​TGA​GCT​GCT​CTT-3′; *WIPI1* forward, 5′-CTT​CAA​GCT​GGA​ACA​GGT​CAC​C-3′, *WIPI1* reverse, 5′-CGG​AGA​AGT​TCA​AGC​GTG​CAG​T-3′; *LAMP1* forward, 5′-ACG​TTA​CAG​CGT​CCA​GCT​CAT-3′, *LAMP1* reverse, 5′-TCT​TTG​GAG​CTC​GCA​TTG​G-3′; *CLCN7* forward, 5′-CCA​CGT​TCA​CCC​TGA​ATT​TTG​T-3′, *CLCN7* reverse, 5′-AAA​CCT​TCC​GAA​GTT​GAT​GAG​G-3′; *CTSD* forward, 5′-GCA​AAC​TGC​TGG​ACA​TCG​CTT​G-3′, *CTSD* reverse, 5′-GCC​ATA​GTG​GAT​GTC​AAA​CGA​GG-3′; *CTSF* forward, 5′-AGA​GAG​GCC​CAA​TCT​CCG​T-3′, *CTSF* reverse, 5′-GCA​TGG​TCA​ATG​AGC​CAA​GG-3′; *Actin* forward, 5′-GTG​GCC​GAG​GAC​TTT​GAT​TG-3′, *Actin* reverse, 5′-AGT​GGG​GTG​GCT​TTT​AGG​ATG-3′.

### Nuclear and cytoplasmic fractionation

Cells were washed twice with PBS and resuspended in lysis buffer (10 mM Hepes, pH 7.4, 10 mM KCl, 1.5 mM MgCl_2_, 0.5 mM DTT) and incubated on ice for 30 min. After 30 s of spin at full speed and then centrifugation at 500 g for 5 min, the supernatant was saved as a cytoplasmic fraction. The pellet was washed twice with lysis buffer and resuspended with nuclear buffer (20 mM Hepes, pH 7.4, 0.4 M NaCl and 1 mM EDTA) containing proteases inhibitors and incubated on ice for 40 min with vortexing for 15 s every 10 min. After centrifugation at 15,000 *g* for 15 min, the supernatant was collected as a nuclear fraction.

### Ubiquitination assay

For analysis of TFEB ubiquitination in vivo, indicated cells were cotransfected with plasmids. Cells were lysed in UREA buffer (50 mM Tris-HCl, pH 8.0, 100 mM NaH_2_PO_4_, 8 M urea, 40 mM imidazole, and 0.5% CHAPS) supplemented with protease inhibitors. Samples were mixed with beads at room temperature for 3 h, and then immunocomplexes were washed three times using lysis buffer, resolved by SDS-PAGE, and analyzed by western blot.

In vitro ubiquitination assays were carried out for 2 h at 37°C in a total volume of 50 μl containing 100 ng purified recombinant GST-TFEB, 100 ng purified recombinant UBA1 (E1), 500 ng UBCH7 (E2), 5 μg purified recombinant WWP2 (E3), and 10 μg ubiquitin in an ATP-regenerating system (50 mM Tris-HCl, pH 7.6, 5 mM MgCl_2_, 2 mM ATP, 10 mM creatine phosphate, 3.5 U/ml creatine kinase, and 0.6 U/ml inorganic pyrophosphatase). After the reaction, the mixture was terminated by the addition of SDS sample buffer and then analyzed by western blot.

### In vitro pull-down assay

Pull-down assay was carried out by incubating purified GST or GST-WWP2 protein with the whole cell lysates overnight at 4°C. The mixture was precipitated using GST beads, followed by further incubation for 2 h at 4°C. The beads were washed and then subjected to western blot.

### Recombinant protein purification and in vitro lactylation assay

GST-tagged TFEB, WWP2, NDRG3, UBA1 (E1) plasmids were made by cloning human TFEB, WWP2, NDRG3, UBA1 into pGEX-5X-1 vector (27-4584-01; GE Healthcare). His-tagged ubiquitin, UBCH7 (E2) plasmids were generated by cloning the corresponding sequence into a pET-32a vector. GST-tagged and His-tagged plasmids were expressed in *Escherichia coli* BL21 (CD601; Transgen Biotech) by induction with 0.1 mM IPTG (PHG0010; Sigma-Aldrich) for 16 h at 18°C to induce protein expression and were harvested at 4,000 × g for 15 min at 4°C and resuspended in lysis buffer (PBS containing 0.5% Triton X-100, 2 mM EDTA, and 1 mM PMSF), followed by ultrasonication. GST-tagged WWP2 and WWP2 inactive mutant were expressed in HEK293T cells. The cells were lysed in NP-40 lysis buffer, followed by ultrasonication. The recombinant GST-tagged proteins were purified using glutathione-Sepharose 4B beads (17-0756-01; GE Healthcare Life Sciences). Then the proteins were eluted with glutathione (S0073; Beyotime) or incubated with TEV protease (a gift from Qiming Sun, Zhejiang University, Hangzhou, China) at 4°C for 4 h to release the proteins from the GST. The His-tagged recombinant proteins were purified using nickel magnetic agarose beads (H9914; Sigma-Aldrich). Purified proteins were eluted under mild conditions by adding 100–250 mM imidazole. Then the glycerol was added to a final concentration of 25% into the eluates for storage at −80°C.

In vitro lactylation assay was performed in 50 μl reaction mixture at 30°C for 1 h. The reaction mixture contained recombinant purified TFEB protein (10 μg) and p300-HA immunoprecipitated from cell lysate, in the presence of lactyl-coenzyme A (4 mg) and 10 μl of 5×HAT assay buffer (250 mM Tris-HCl, pH 8.0, 5 mM dithiothreitol, 50% glycerol, 0.5 mM EDTA). Then the reaction system was stopped by the addition of 4× sample buffer and probed with anti-Kla.

### Immunofluorescence and immunohistochemistry

Cells were cultured on coverslips and fixed in 4% formaldehyde for 15 min at room temperature. After washing three times with PBS, cells were permeabilized with 0.1% Triton X-100 in PBS for 10 min. Cells were incubated in 10% FBS in PBS for 1 h and then with primary antibodies in 10% FBS for 3 h at 37°C. Cells were washed three times with PBS and incubated with secondary antibodies conjugated to Donkey-anti-Mouse-Alexa Fluor546 (A10036; Invitrogen), Donkey-anti-Rabbit-Alexa Fluor488 (A21206; Invitrogen) in 10% FBS for 2 h at 37°C. The washed coverslips were mounted in vector shield and observed using Meta laser-scanning confocal microscope 880 (Carl Zeiss) with Plan-Apochromat 63× oil immersion objective (NA1.4). GFP-LC3 and Cherry-LC3 puncta with diameters between 0.3 and 1 μm were scored as positive. Images were acquired using ZEN (black edition) 2.3 software.

The clinical tissue samples were fixed overnight in 4% paraformaldehyde and then embedded in paraffin. Histologic sections for light microscopy were cut to a thickness of 3 μm. Unstained slides were baked at 60°C for 2 h, deparaffinized in xylenes (three times, 10 min each), and rehydrated sequentially in ethanol (5 min in 100%, 2 min in 95%, 2 min in 85%, 2 min in 75%, 2 min in 50%, 2 min in water). For antigen unmasking, specimens were boiled in 1× sodium citrate buffer and rinsed three times with PBS. Endogenous peroxidase activity in tissue sections was blocked by treatment with 1% H_2_O_2_ at room temperature for 10 min, washed three times with PBS, and then blocked with 5% goat serum in PBS for 45 min. Anti-lactyl-lysine (1:100) was diluted in blocking solution and incubated with the tissue sections at 4°C overnight. This was followed by incubation with goat anti-rabbit HRP secondary antibody for 1 h at room temperature. The immunostained sections were then exposed to a DAB substrate kit (SK-4100), washed with water, and counterstained with hematoxylin. In addition, the corresponding fluorescent secondary antibodies were also used. Stained slides were visualized using a Pannoramic MIDI (3D Histech) with 40× objective. Images were quantified using ImageJ software.

### RNA interference

Cells were transfected with specific siRNAs using Lipofectamine 2000 for 72 h. The following siRNA duplexes were used: TFEB siRNA: 5′-AAA​CGG​AGC​CUA​CUG​AAC​A-3′; LDHA siRNA #1: 5′-GGC​AAA​GAC​UAU​AAU​GUA​A-3′; LDHA siRNA #2: 5′-UUG​UUG​AUG​UCA​UCG​AAG-3′; LDHA siRNA #3: 5′-GGG​UCC​UUG​GGG​AAC​AUG-3′; MCT1 siRNA: 5′-AAG​AGG​CUG​ACU​UUU​CCA​AAU-3′; MCT4 siRNA: 5′-CGA​CCC​ACG​UCU​ACA​UGU​ACG​UGU​U-3′; WWP2 siRNA: 5′-UGA​CAA​AGU​UGG​AAG​GAA​UU-3′; STUB1 siRNA#1: 5′-GGC​AAU​CGU​CUG​UUC​GUG​GGC​CGA​A-3′; STUB1 siRNA#2: 5′-GGC​AGU​CUG​UGA​AGG​CGC​ACU​UCU​U-3′; PDHA siRNA: 5′-GGU​CAG​AUC​UUU​GAA​GCU​U-3′; Non-targeting siRNA: 5′-UUC​UCC​GAA​CGU​GUC​ACG​U-3′.

### HPLC-MS/MS

To identify the interaction proteins of TFEB, HEK293T cells transiently expressing TFEB-Flag were immunoprecipitated with anti-Flag beads. Then the beads were washed four times with Tris-HCl (100 mM, pH 8.5) and then dissolved with urea (8 M). The mixture was sonicated for 30 min at room temperature, and then tris(2-carboxyethyl) phosphate (final concentration is 5 mM; Thermo Fisher Scientific) and iodoacetamide (final concentration is 10 mM) (Sigma-Aldrich) were added to the solution and incubated at room temperature for 20 and 15 min for reduction and alkylation, respectively. The solution was diluted four times and digested with Trypsin at 1:50 (wt/wt) (Promega).

To identify the lactylation site of TFEB by MS, HeLa cells were transfected with TFEB-Flag and then treated with lactate. Immunoprecipitation was performed 24 h after transfection with anti-Flag affinity beads and then separated by SDS-PAGE and stained with colloidal Coomassie blue staining. The gel band of TFEB-Flag was cut into small pieces. In-gel digestion of TFEB was performed using trypsin overnight at 37°C. The tryptic-digested peptides were then desalted by C18-monospin column for further analysis.

For MS analysis, the tryptic digested peptides were loaded on a capillary reverse-phase C18 column packed in-house (150 mm length, 1.9 mm particle size, 75 μm ID × 360 μm OD, 100 Å pore diameter) connected to an Easy LC 1000 system (Thermo Fisher Scientific) for MS analysis. Data-dependent tandem MS (MS/MS) analysis was performed with a Q Exactive Orbitrap mass spectrometer (Thermo Fisher Scientific). Peptides eluted from the LC system were directly electrosprayed into the mass spectrometer with a distal 1.8-kV spray voltage. One acquisition cycle includes one full-scan MS spectrum (m/z 300–1,800) with resolution r = 70,000 at m.z 400 followed by top 20 MS/MS events, sequentially generated on the first to the twentieth most intense ions selected from the full MS spectrum at a 27% normalized collision energy. MS scan functions and LC solvent gradients were controlled by the Xcalibur data system (Thermo Fisher Scientific). The samples were analyzed with a 180 min-HPLC gradient from 0 to 100% of 0.1% formic acid in acetonitrile at 300 nl/min.

The acquired MS/MS data were analyzed against a UniProtKB *Homo sapiens* database using PEAKS Studio (BSI.Inc.). To accurately estimate peptide probabilities and false discovery rates, we used a decoy database containing the reversed sequences of all the proteins appended to the target database. Carbamidomethylation (+57.02146) of cysteine was considered as a static modification and lysine lactylation (+72.02) of K as a variable modification.

### Measurement of intracellular lactate levels and pH value

Intracellular lactate level was measured by using the lactate Colorimetric/Fluorometric Assay Kit (K627-100; Biovision) according to the manufacturer’s protocol.

To measure intracellular pH, cells were incubated with 10 μM cell-permeable probe 5,6-carboxy-SNARF-1 acetoxymethyl ester acetate (SNARF-1-AM) for 30 min at 37°C. After being washed twice with HBSS, cells were trypsinized and washed with HBSS. Fluorescence emission at 580 and 642 nm after excitation at 484 nm was recorded by flow cytometry using the Cytomic FC 500 MCL. The standard curve was generated by treating cells with 10 μM SNARF-1-AM in calibration buffers from pH 3.5–7.5 (Invitrogen). The fluorescence ratio 642/580 was converted to pH values using the standard curve.

### LysoTracker staining

Cells were cultured on coverslips and incubated with 50 nM LysoTracker red (L-7528; Life Technologies) for 30 min at 37°C. After being washed twice with PBS, cells were visualized by using Meta laser-scanning confocal microscope 880 (Carl Zeiss) with Plan-Apochromat 63× oil immersion objective (NA1.4). Acidic vesicles were counted manually using ImageJ software analysis.

### Isothermal titration calorimetry (ITC) binding assay

Thermodynamic parameters of the binding of lactate to TFEB or NDRG3 were measured using the VP-ITC microcalorimeter (Malvern Instruments Ltd) at 25°C. In all experiments, the initial injection of 2 μl of lactate was discarded to eliminate the effect of titrant diffusion across the syringe tip during the equilibration process, and each dataset consisted of 25 injections of 5 μl each of 10 mM lactate into a sample cell containing 250 μl of 0.5 mM TFEB or NDRG3. The raw data were processed using the single binding site model in the MicroCal ORIGIN software.

### Intrinsic tryptophan fluorescence binding assay

The binding of lactate to TFEB and NDRG3 was examined by measuring the fluorescence quenching of TFEB and NDRG3 tryptophan residues after addition of lactate ligand. The intrinsic tryptophan fluorescence of TFEB and NDRG3 (100 μM in 10 mM NaH_2_PO_4_, pH 7.5) was monitored from 300 to 400 nm after excitation at 295 nm (to minimize interference from tyrosine fluorescence) both before and after addition of increasing increments of lactate using a Thermo Fisher Scientific 5250040. The intrinsic tryptophan fluorescence in the presence of different concentrations of lactate was plotted as the maximum fluorescence difference (ΔF = F_0_–F) versus ligand concentration to yield a saturation curve. F_0_ and F were the measured fluorescence of the solution in the absence and presence of lactate, respectively.

### Human PDA tissues

The 18 paired human PDA patient samples were collected from The Second Affiliated Hospital of Zhejiang University School of Medicine. The detailed information is supplied in Table S2. The use of these clinical samples has been approved by the Second Affiliated Hospital of Zhejiang University School of Medicine.

### Colony formation assay

Cells were seeded in 6-well plates at 1,000 cells per well in 2 ml of medium and incubated at 37°C for 2 wk. Colony plates were fixed in 4% paraformaldehyde and stained with 0.1% crystal violet.

### Statistical analysis

Unpaired two-sided *t* tests were used for comparisons between two groups. Ordinary one-way analysis of variance (ANOVA) followed by post hoc Tukey’s multiple comparison were used for three or more groups. Data distribution was assumed to be normal, but this was not formally tested. Statistical analyses were conducted using an unpaired *t* test, *P < 0.05 was considered statistically significant. All the statistical data are presented as mean ± SEM. Data were analyzed by GraphPad Prism 5 software.

### Online supplemental material

The associated supplemental files contain six figures and two tables. Fig. S1 is related to Fig. 1, showing that lactate promotes autophagy and lysosome biogenesis. Fig. S2 is related to Fig. 2, showing the effect of lactate on TFEB expression. Fig. S3 is related to Figs. 3 and 4, showing that lactate inhibits TFEB ubiquitination and proteasomal degradation. Fig. S4 is related to Fig. 5, showing the identification of TFEB lactylation at K91. Fig. S5 is related to Fig. 6, showing that lactylation prevents TFEB-WWP2 interaction and TFEB degradation. Table S1 contains the MS data of TFEB binding proteins identified by HPLC-MS/MS. Table S2 contains the clinical data of 18 PDA patients included in this study.

## Supplementary Material

Table S1 shows TFEB binding proteins identified by HPLC-MS/MS.

Table S2 shows clinical data of 18 PDA patients included in this study.

SourceData F1 is the source file for Fig. 1.

SourceData F2 is the source file for Fig. 2.

SourceData F3 is the source file for Fig. 3.

SourceData F4 is the source file for Fig. 4.

SourceData F5 is the source file for Fig. 5.

SourceData F6 is the source file for Fig. 6.

SourceData F7 is the source file for Fig. 7.

SourceData FS1 is the source file for Fig. S1.

SourceData FS2 is the source file for Fig. S2.

SourceData FS3 is the source file for Fig. S3.

SourceData FS4 is the source file for Fig. S4.

SourceData FS5 is the source file for Fig. S5.

## Data Availability

The MS proteomics data have been deposited to the ProteomeXchange Consortium (https://proteomecentral.proteomexchange.org) via the iProX partner repository (Chen et al., 2022; Ma et al., 2019) with the dataset identifier PXD050578. Other original data, strains, and plasmids generated in this study are available within the article and its supplementary data files, or from the corresponding author upon request.
