# Supplementary figures and images for: Lactylation stabilizes TFEB to elevate autophagy and lysosomal activity
Source: J Cell Biol. 2024 Aug 28;223(11):e202308099. doi: 10.1083/jcb.202308099 (PMC11354204; doi:10.1083/jcb.202308099)

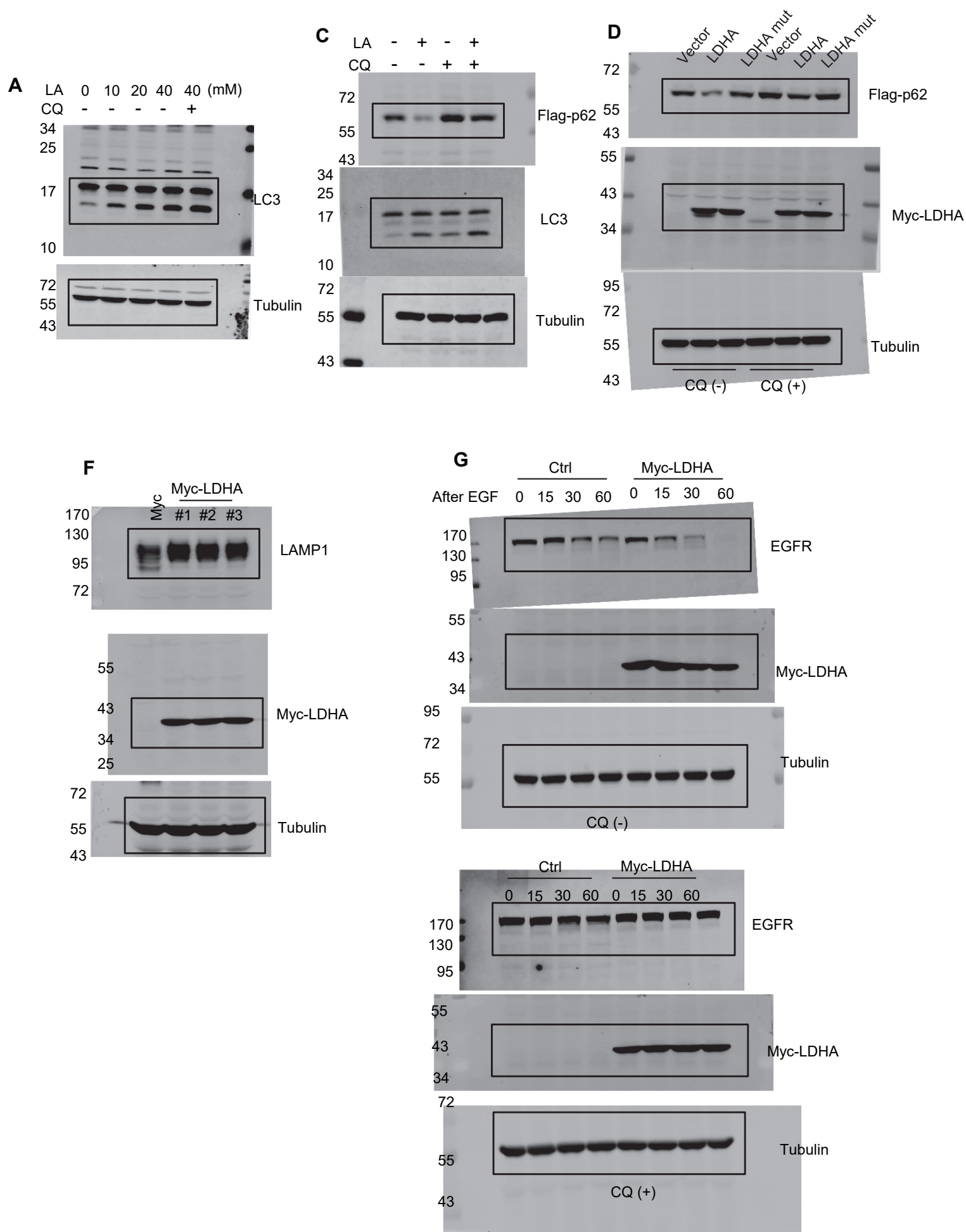

Figure 1

Supplement: SourceData F1 — is the source file for Fig. 1. [file JCB_202308099_SourceDataF1.pdf]

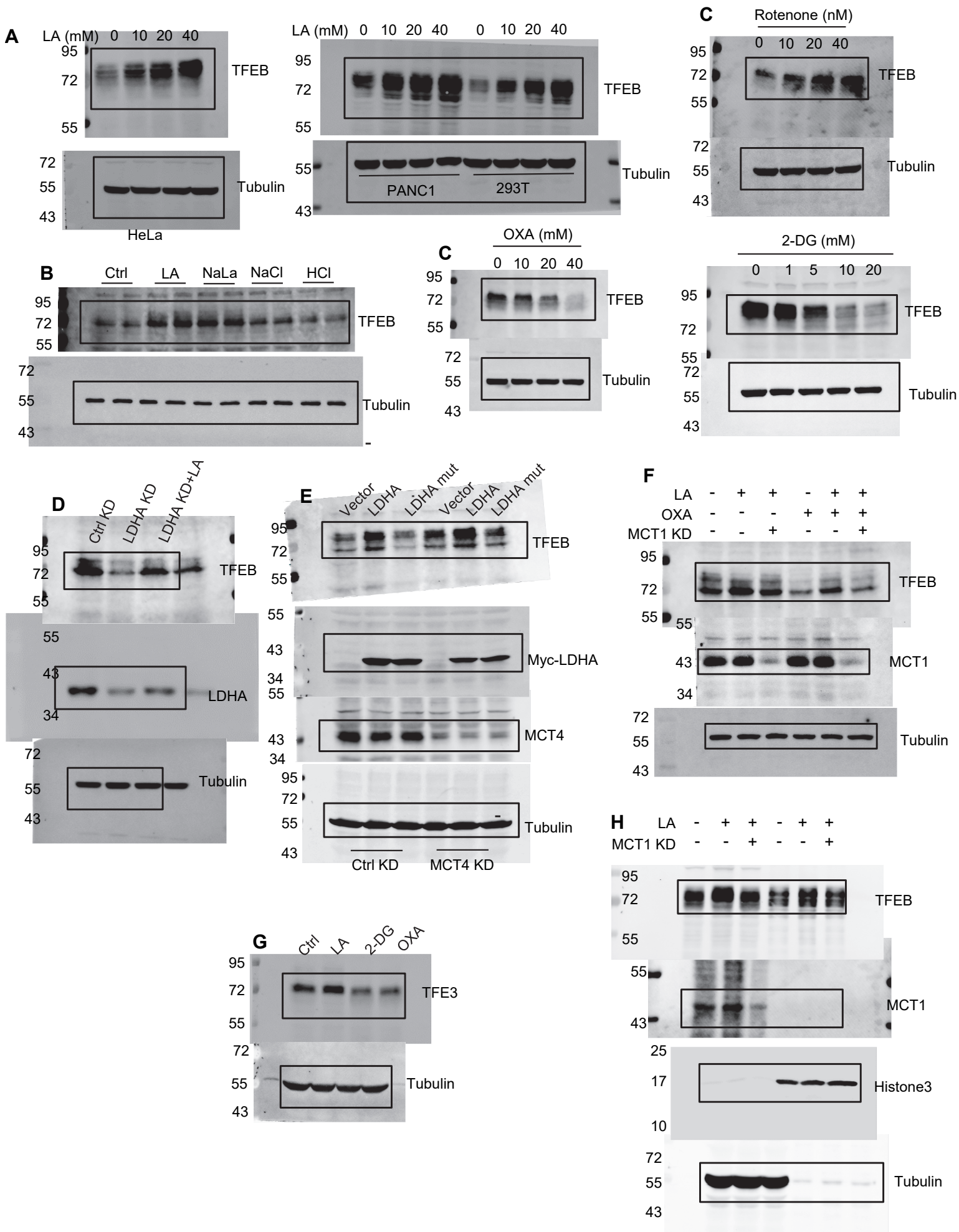

Figure 2

Supplement: SourceData F2 — is the source file for Fig. 2. [file JCB_202308099_SourceDataF2.pdf]

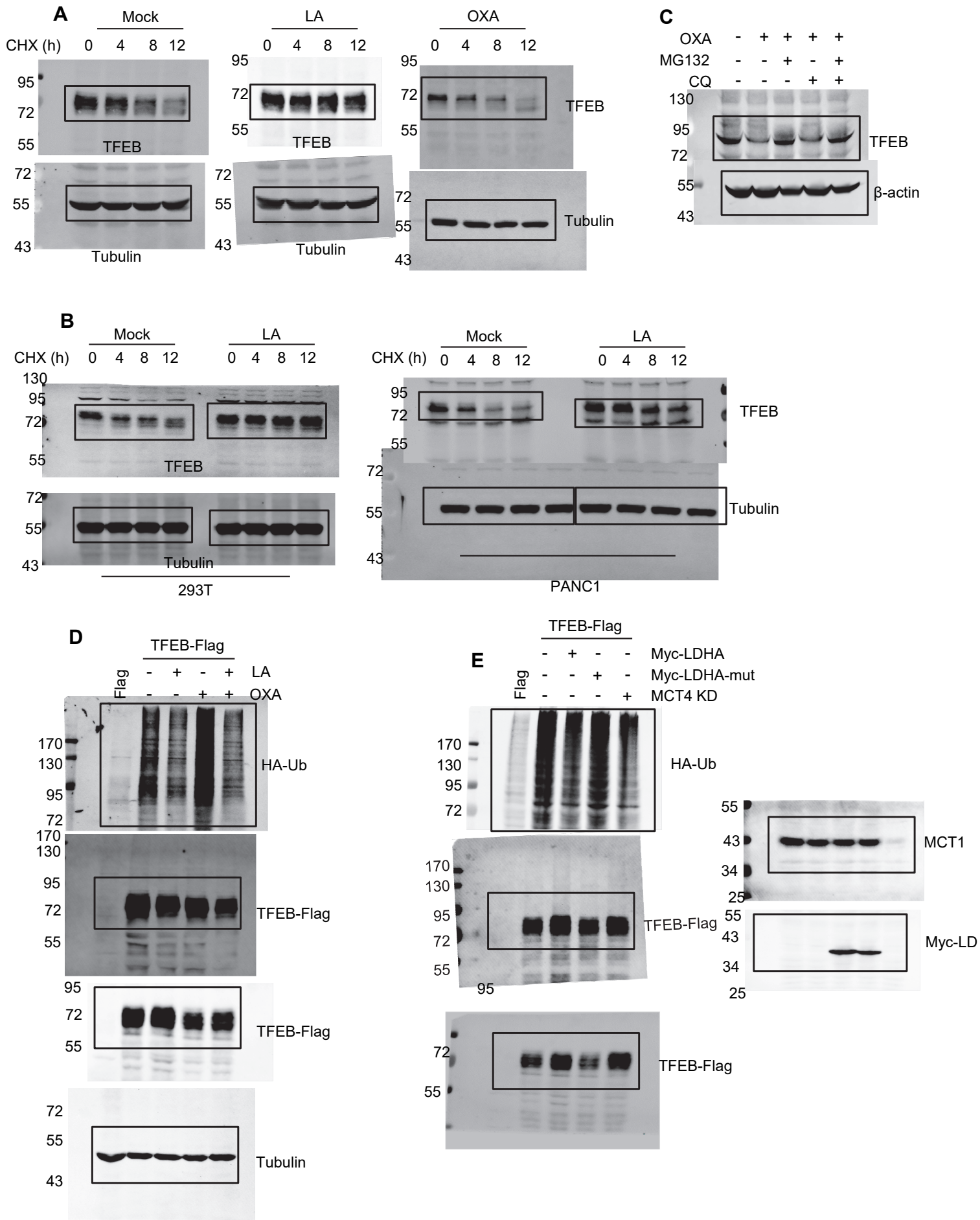

Figure 3

Supplement: SourceData F3 — is the source file for Fig. 3. [file JCB_202308099_SourceDataF3.pdf]

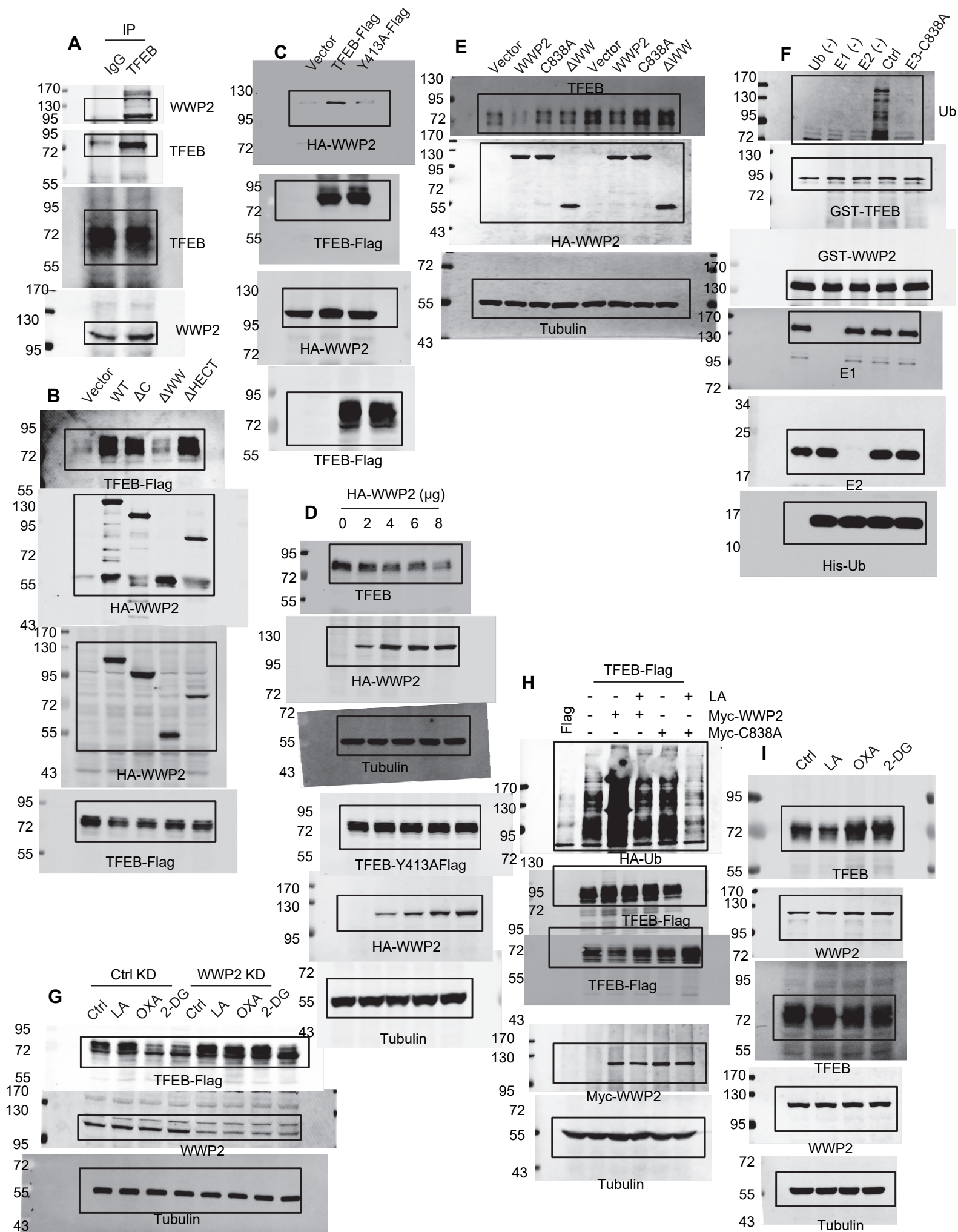

Figure 4

Supplement: SourceData F4 — is the source file for Fig. 4. [file JCB_202308099_SourceDataF4.pdf]

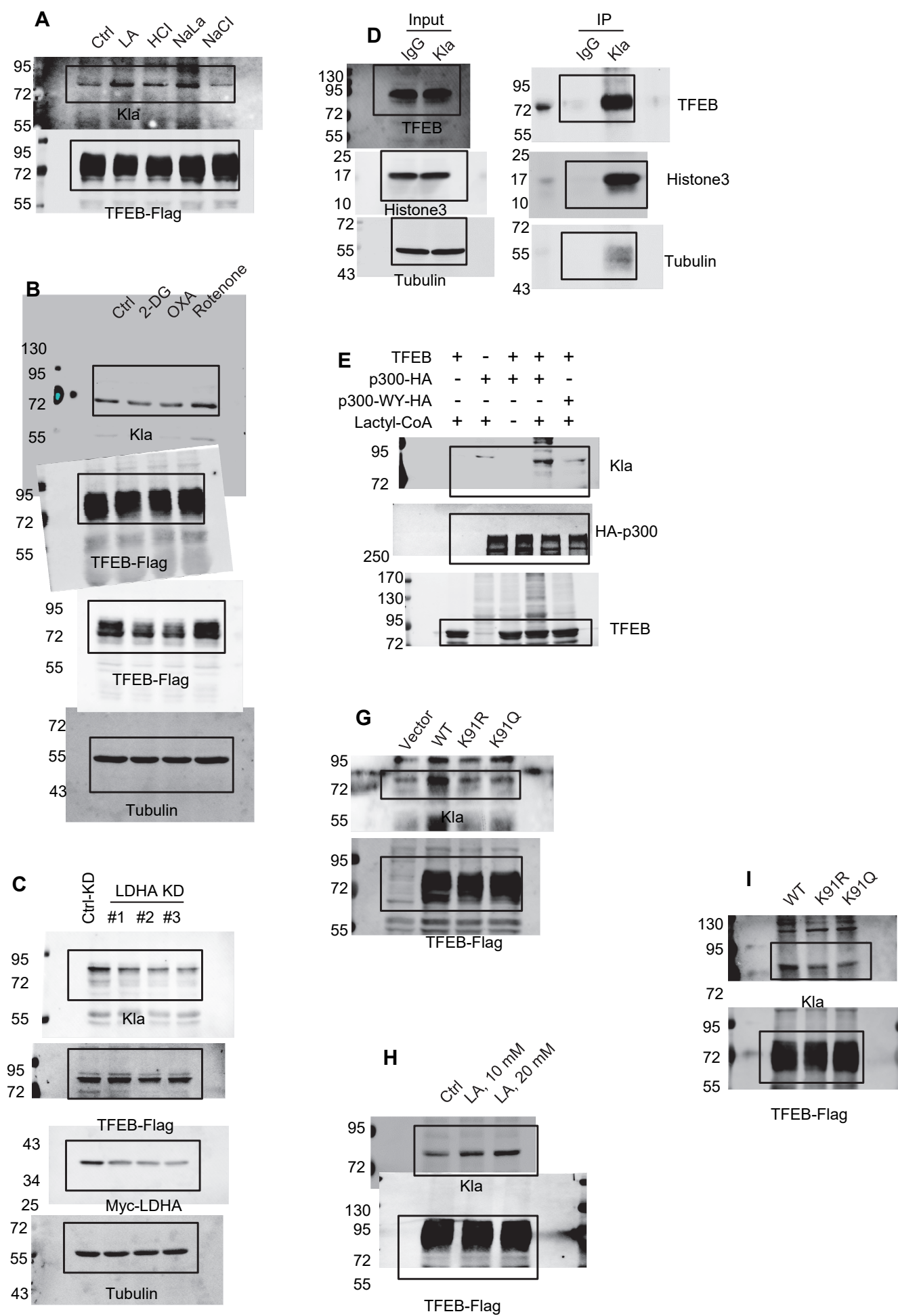

Figure 5

Supplement: SourceData F5 — is the source file for Fig. 5. [file JCB_202308099_SourceDataF5.pdf]

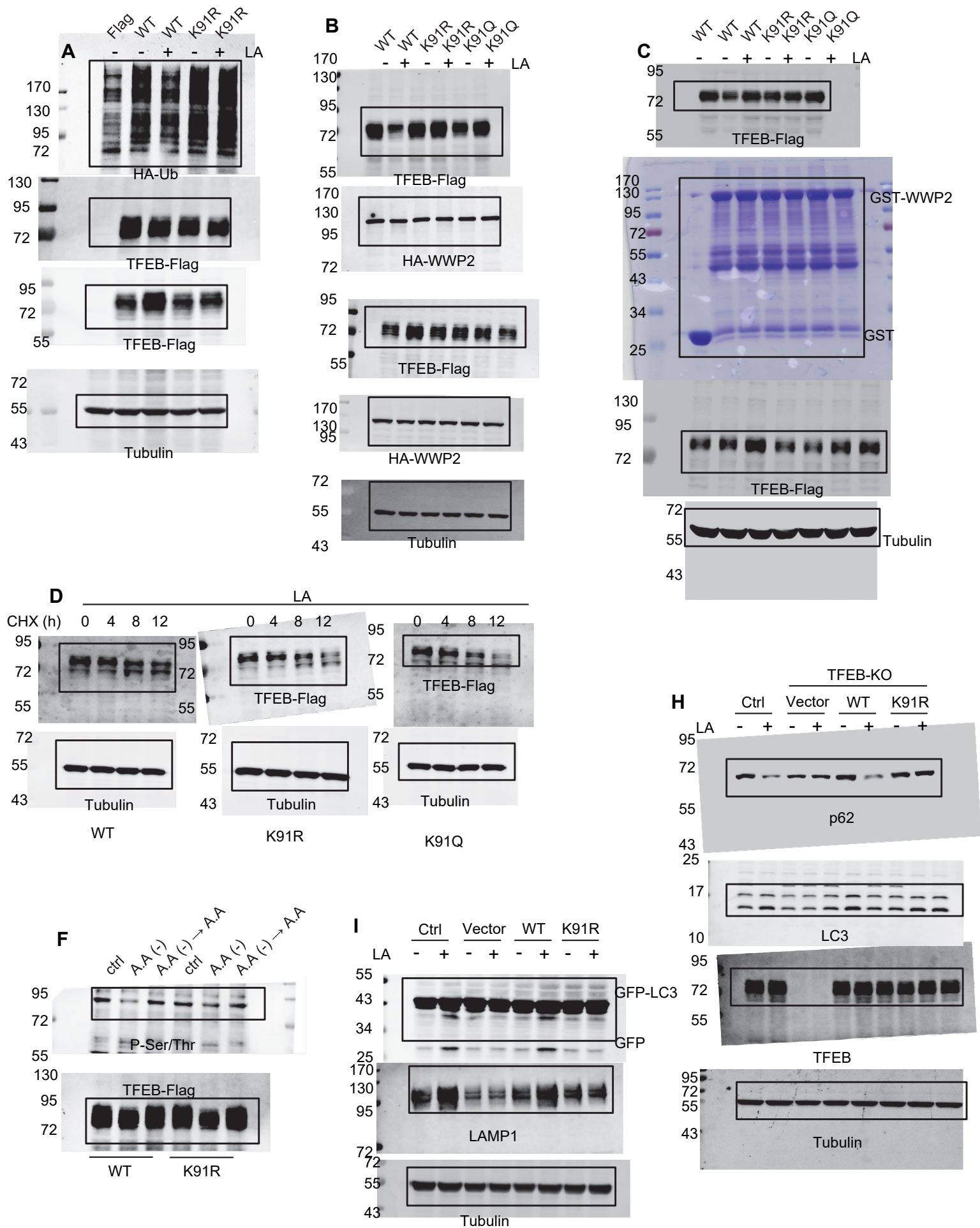

Figure 6

Supplement: SourceData F6 — is the source file for Fig. 6. [file JCB_202308099_SourceDataF6.pdf]

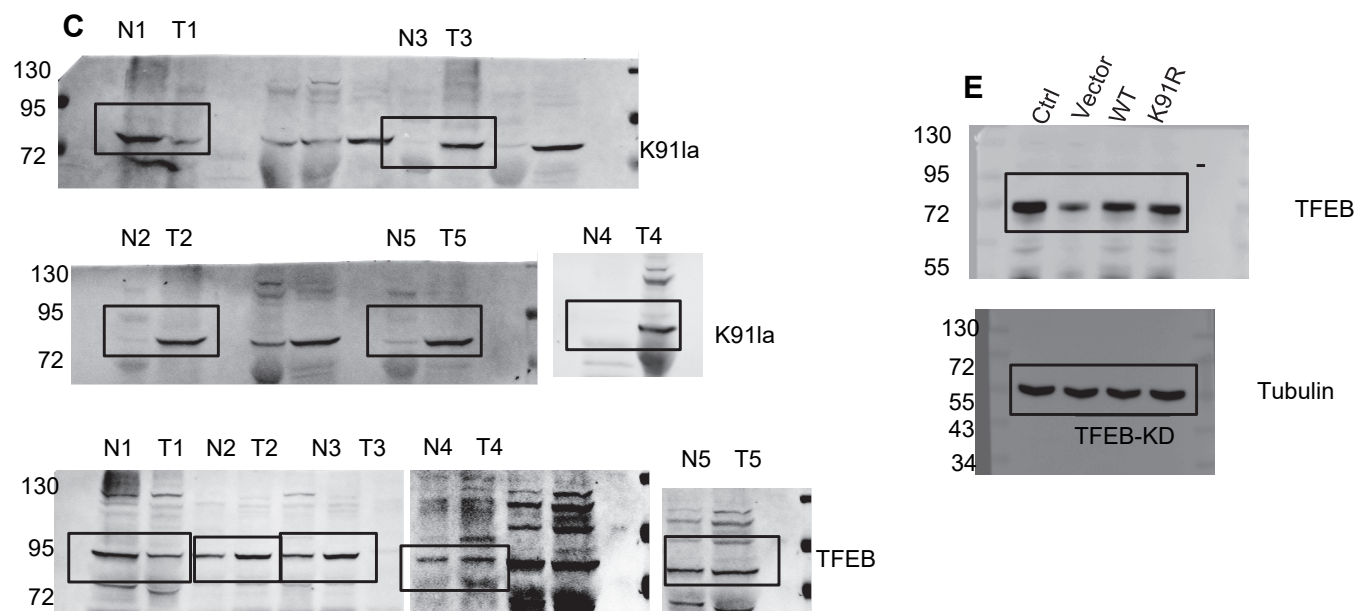

Figure 7

Supplement: SourceData F7 — is the source file for Fig. 7. [file JCB_202308099_SourceDataF7.pdf]

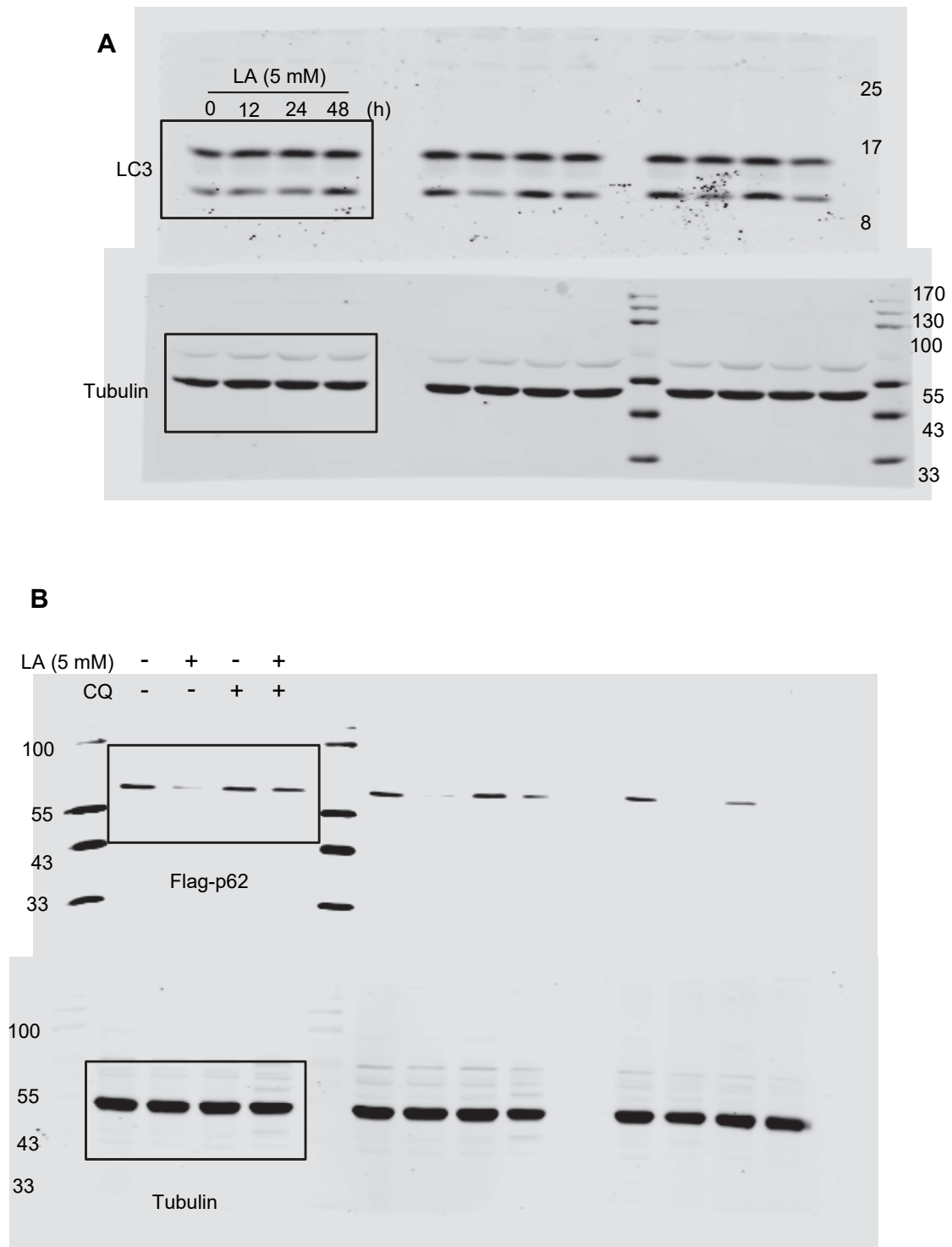

Figure S1

Supplement: SourceData FS1 — is the source file for Fig. S1. [file JCB_202308099_SourceDataFS1.pdf]

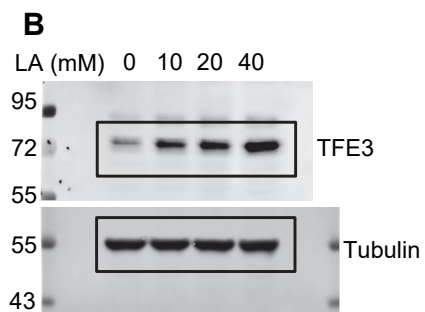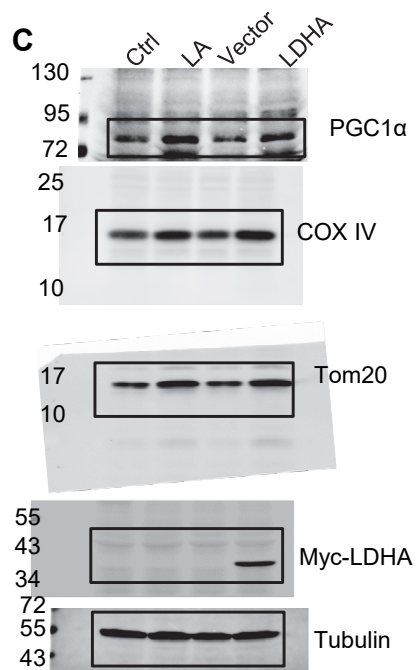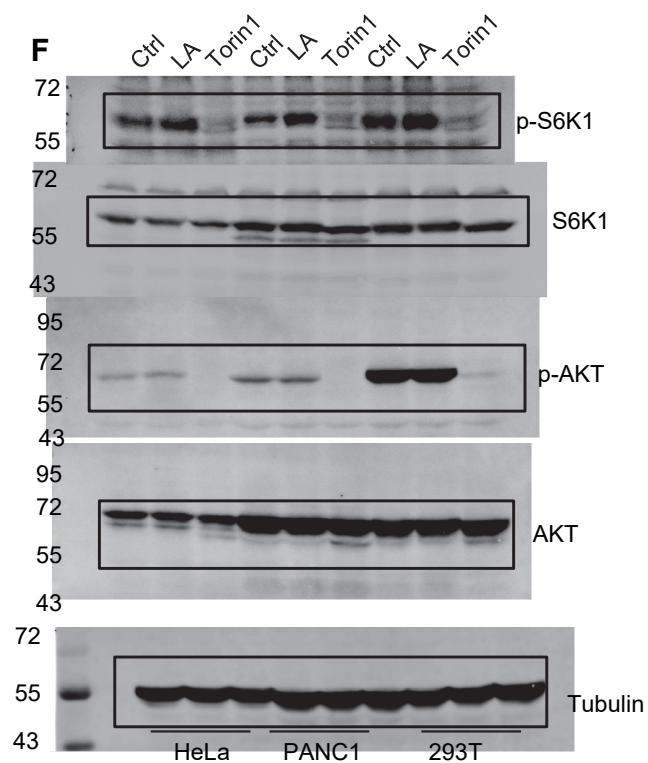

Figure S2

Supplement: SourceData FS2 — is the source file for Fig. S2. [file JCB_202308099_SourceDataFS2.pdf]

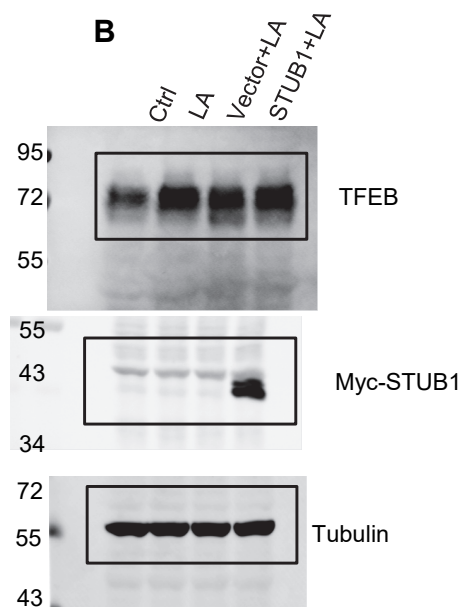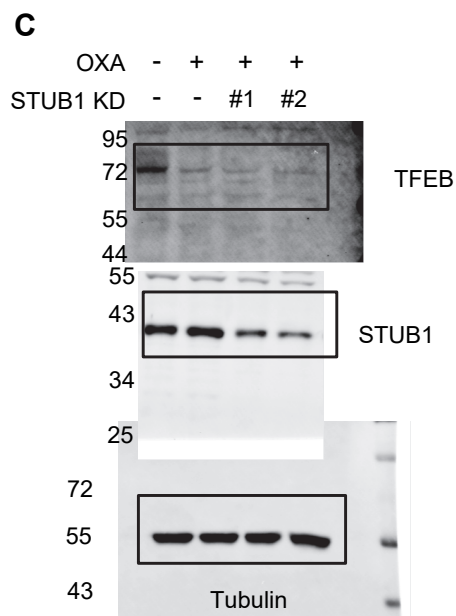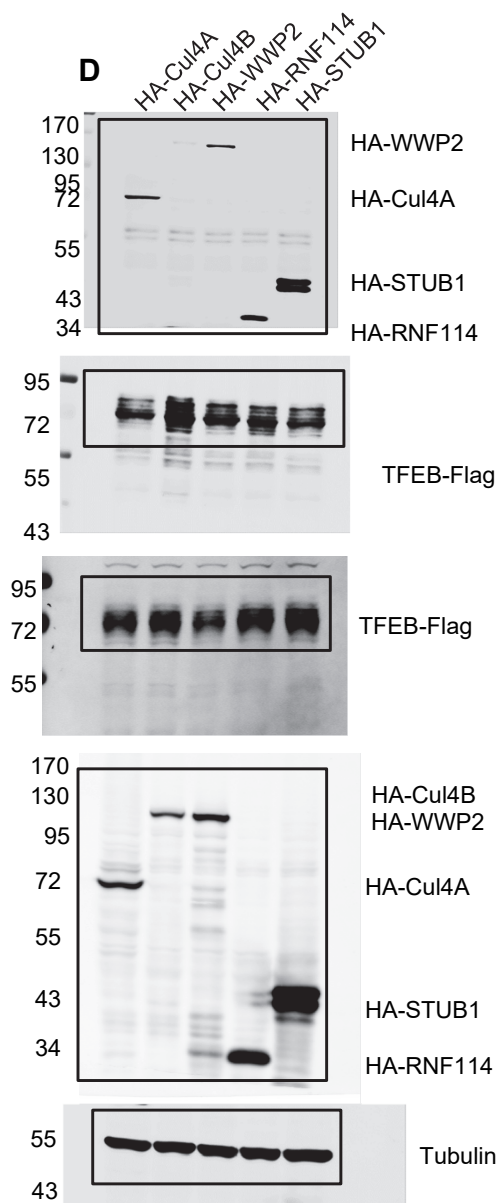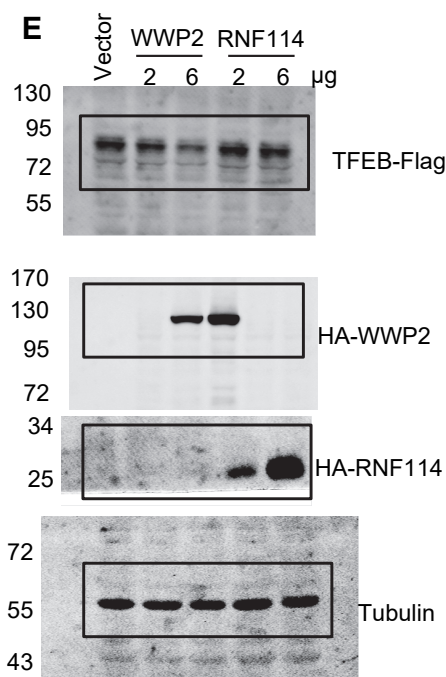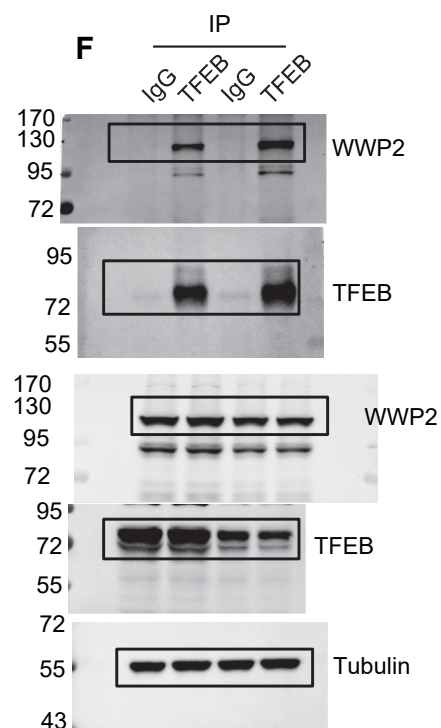

Figure S3

Supplement: SourceData FS3 — is the source file for Fig. S3. [file JCB_202308099_SourceDataFS3.pdf]

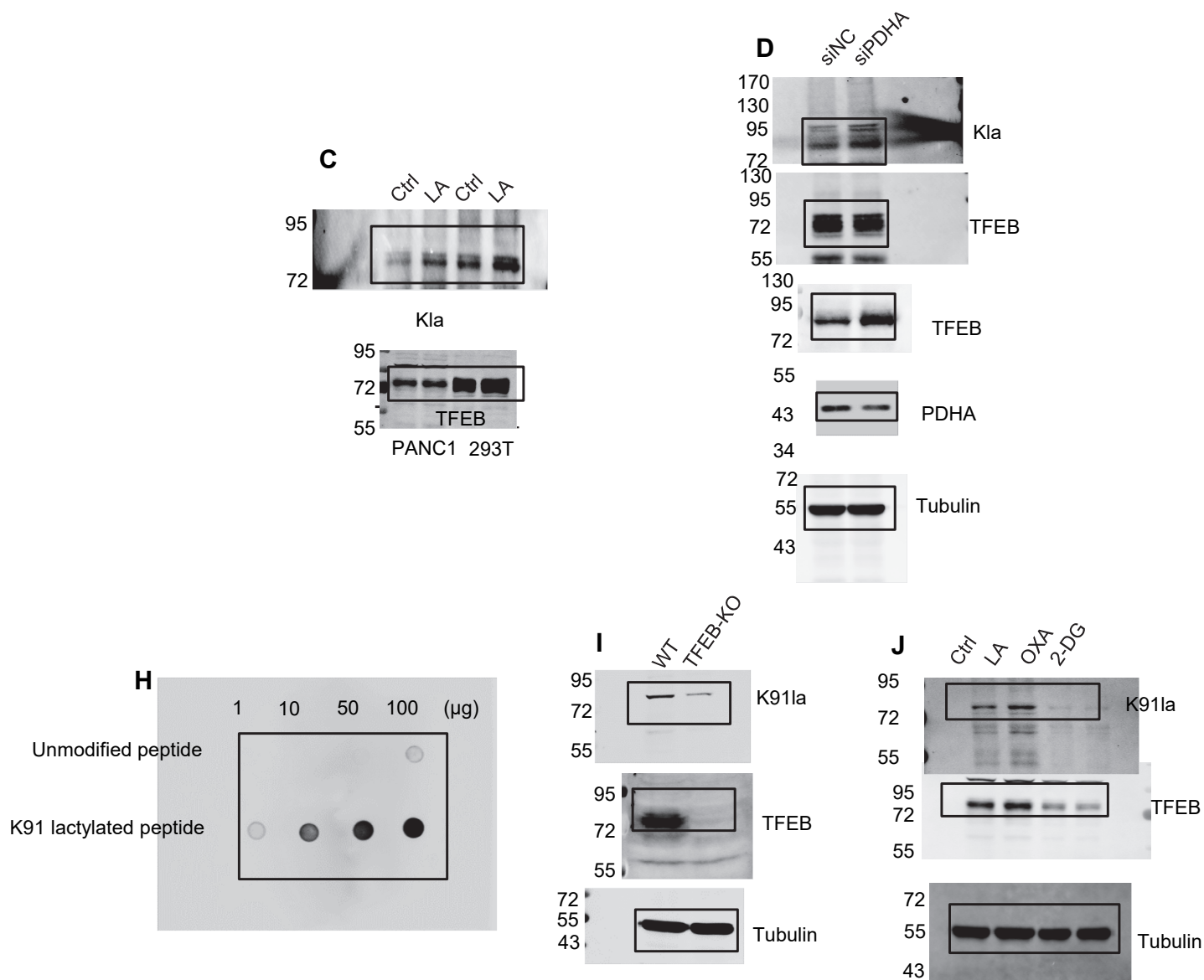

Figure S4

Supplement: SourceData FS4 — is the source file for Fig. S4. [file JCB_202308099_SourceDataFS4.pdf]

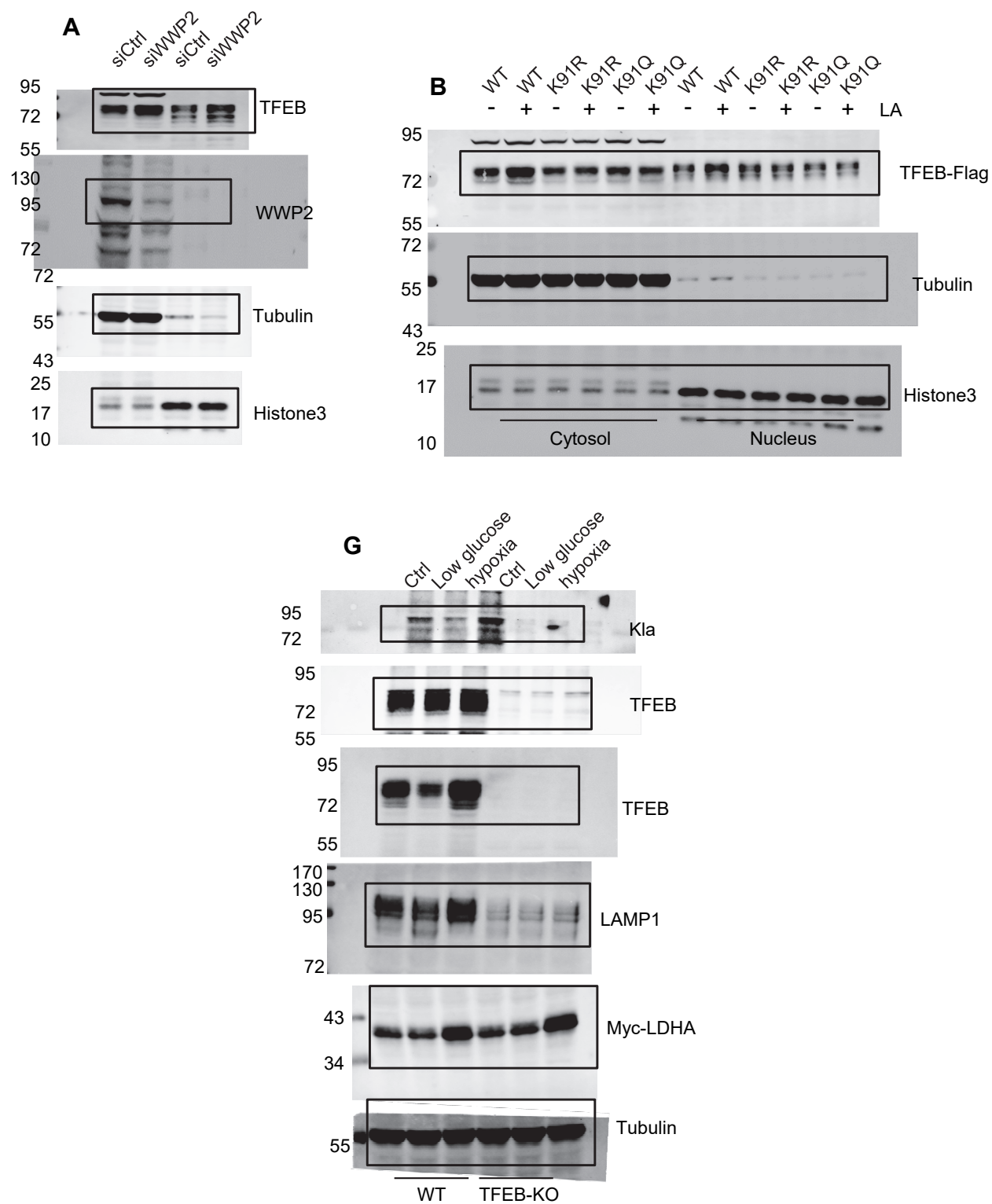

Figure S5

Supplement: SourceData FS5 — is the source file for Fig. S5. [file JCB_202308099_SourceDataFS5.pdf]
